# Melanoma-specific antigen-associated antitumor antibody reactivity as an immune-related biomarker for targeted immunotherapies

**DOI:** 10.1038/s43856-022-00114-7

**Published:** 2022-05-11

**Authors:** Annika Rähni, Mariliis Jaago, Helle Sadam, Nadežda Pupina, Arno Pihlak, Jürgen Tuvikene, Margus Annuk, Andrus Mägi, Tõnis Timmusk, Amir M. Ghaemmaghami, Kaia Palm

**Affiliations:** 1 Protobios Llc, Tallinn, Estonia; 2 Department of Chemistry and Biotechnology, Tallinn University of Technology, Tallinn, Estonia; 3 dxlabs Llc, Tallinn, Estonia; 4 EGeen International Inc., Mountain View, CA USA; 5 Tartu University Hospital, Tartu, Estonia; 6 Immunology and Immuno-Bioengineering Group, School of Life Science, Faculty of Medicine and Health Sciences, University of Nottingham, Nottingham, UK

**Keywords:** Prognostic markers, Tumour immunology, Melanoma

## Abstract

**Background:**

Immunotherapies, including cancer vaccines and immune checkpoint inhibitors have transformed the management of many cancers. However, a large number of patients show resistance to these immunotherapies and current research has provided limited findings for predicting response to precision immunotherapy treatments.

**Methods:**

Here, we applied the next generation phage display mimotope variation analysis (MVA) to profile antibody response and dissect the role of humoral immunity in targeted cancer therapies, namely anti-tumor dendritic cell vaccine (MelCancerVac^®^) and immunotherapy with anti-PD-1 monoclonal antibodies (pembrolizumab).

**Results:**

Analysis of the antibody immune response led to the characterization of epitopes that were linked to melanoma-associated and cancer-testis antigens (CTA) whose antibody response was induced upon MelCancerVac® treatments of lung cancer. Several of these epitopes aligned to antigens with strong immune response in patients with unresectable metastatic melanoma receiving anti-PD-1 therapy.

**Conclusions:**

This study provides insights into the differences and similarities in tumor-specific immunogenicity related to targeted immune treatments. The antibody epitopes as biomarkers reflect melanoma-associated features of immune response, and also provide insights into the molecular pathways contributing to the pathogenesis of cancer. Concluding, antibody epitope response can be useful in predicting anti-cancer immunity elicited by immunotherapy.

## Plain language summary

Immunotherapy treatments, which utilize the patient’s own immune system to fight cancer, have become a standard treatment of cancer. However, for many patients’ immunotherapy does not work. During the immune response the body produces proteins called antibodies. This study characterized the antibodies produced following treatment with two different types of immunotherapies that treat skin cancer, to gain insights into how the immune system responds in different individuals. Our results demonstrate that multiple proteins that are present in patients with skin cancer are specifically targeted by the immune system during skin cancer specific immunotherapy. Our results should help further anti-cancer drug development.

## Abstract (web summary)

Rähni et al profile antibody response in patients with varied response to cancer immunotherapies. They identify antibody epitope responses that predict anti-cancer immunity elicited by immunotherapy.

## Introduction

Knowledge of the immunosuppressive tumor microenvironment has markedly improved within the last decade (reviewed in ref. ^1^). To achieve immunogenicity, tumor cells must express antigens capable of eliciting immune activation. The identification of applicable tumor antigens is indispensable for the development of effective cancer immunotherapy. Most known tumor antigens are considered canonical if derived from protein-coding regions in contrast to noncanonical antigens that include sequences outside protein-coding regions or that are generated by antigen-processing^2^. Melanoma cells are considered highly immunogenic with well-described tumor-associated antigens (TAAs)^3^, including cancer-testis antigens (CTAs)^4^ and neo-antigens carrying novel epitopes of self-antigens^5^. Some well-known examples include carcinoembryonic antigen (CEA), B melanoma antigen 1 (BAGE), G antigens (GAGEs), cancer/testis antigen 1 (CTAG1; also known as NY-ESO1), and melanoma-associated antigens (MAGEs) (Rev in ref. ^6^). The antigenic repertoire is a critical factor for immunosurveillance and cancer progression^7^. However, most studies have focused on the role of T cells in these battles^8^, while considerably less is known about B cell response^9^. Humoral response against cross-reactive autoantigens has been detected in different cancers^10^. A burst of recent publications is pointing to the role of antibodies contributing to tumor control^11^ as cancer-associated autoimmunity targeting non-malignant tissues may reflect favorable disease outcome^12^. On the other hand, the reasons underlying the immunogenicity of the tumor, or the lack of it, are not well understood^13^. The antitumor immunity can result from many factors including MHC genetic variation, tumor mutational load, tissue microenvironment^13^, but also by cell stress, reactivation of embryonic or gonadal transcription, epigenetic instability, aberrant RNA splicing, and others^14,15^. For example, it is argued that the capture of either apoptotic or necrotic cancer cells by macrophages and dendritic cells in the tumor microenvironment may lead to immune suppression or stimulate inflammatory pathways contributing to antitumor cytotoxicity^16^.

Discoveries in cancer biology have led to new strategies in awakening tumor immunogenicity, including checkpoint blockade, adoptive cellular therapy, and cancer vaccines, underscoring the role of the immune system in waging the war on cancer tissue. Among these are monoclonal antibodies that target cancer immune checkpoint inhibitors (ICIs) including anti-CTLA-4, anti-PD-1, and anti-PD-L1/2 antibodies that are able to restore anticancer immunity and are widely used for the management of various cancers, including melanoma^17^. Immunogenicity of CTAs has led to the use of melanoma-associated antigens as promising candidates for novel cancer treatments^18,19^. In addition to monoclonal antibodies, cancer vaccines, in particular those based on dendritic cells (DCs) as vectors for antigen delivery, are a major focus of current developments^20^. To date, personalized neoantigen-based DC vaccines are evolving and have shown clinical success in melanoma and other solid tumors^21^.

Biomarkers associated with clinical prognosis of the cancer and/or severe immune-related adverse effects (irAEs) of the drugs are areas of active investigation. Different biomarkers have been tackled with variable success, such as levels of PD-L1^22^, genetic mutations^23^, inflammatory cytokines^24^, and the presence of tumor-infiltrating lymphocytes (reviewed in ref. ^25^). Tumor infiltrating B lymphocytes contribute to anti-tumor immunity by promoting antibody response to tumor antigens^26,27^. High titer antibodies against melanoma differentiation antigens (TRP1/TYRP1, TRP2/TYRP2, gp100, MelanA/MART1) were observed in responder group of melanoma patients treated with ICI mAbs (monotherapies with Nivolumab, Pembrolizumab or Ipilimumab, or the combination of Nivolumab and Ipilimumab)^28,29^. However, pre-treatment autoantibody profiles in melanoma patients were reported to predict ICI treatment-associated toxicity^30^. Connectedly, DC vaccines also stimulate robust antibody response^31–33^ and in some cases, this is associated with prolonged recurrence-free survival^32^. Despite big hopes, clinical benefit of immunotherapies has remained limited only to a subset of patients^34,35^ and it is currently undetermined whether increase or decrease in immune response to specific tumor antigens is beneficial to the patient^36,37^.

Here, we explore the use of a high precision approach called mimotope variation analysis (MVA), a next generation random peptide phage display method to delineate cancer therapy-associated antibody immune response at epitope resolution. We hypothesize that the pre-existing and treatment-induced antibodies against specific antigen targets could reflect the response elicited by anti-tumor drug and that this response could be predictive of cancer immunogenicity and thus, sensitivity to immune therapy. We generate data to test this hypothesis by immunoprofiling analysis of the anti-melanoma antibody response in the sera samples from the phase II clinical trial of patients with non-small cell lung cancer (NSCLC) receiving autologous DC therapy based on allogenic melanoma cell lysate (MelCancerVac^®^)^38,39^. We correlate the findings on melanoma-specific antigen profiles with those from a group of patients with unresectable metastatic melanoma receiving anti-PD1 (pembrolizumab) treatment as a part of their standard-of-care. We verify the melanoma-antigen specificity using MVA-based competition, and further determine a three-epitope biomarker signature of melanoma-specific antibody response elicited by both immunotherapies. Our results demonstrate the relevance of antibody epitope profiling to better understand the fine line separating beneficial immunosurveillance from harmful autoimmunity in the anticancer immune response elicited by different types of therapy.

## Methods

### Study population

The present study analyzed samples from a total of 119 individuals from 2 different clinical cohorts of NSCLC and melanoma patients and their appropriate controls, whose clinical characteristics are shown in Table 1 and Supplementary Table 1. The study was conducted in accordance with the guiding principles of the Declaration of Helsinki and the study participants gave informed consent before enrollment.

**Table 1:** Description of clinical cohorts.

Cohort	Cohort (*n* = 119 individuals)				
Sub-cohort	NSCLC sub-cohort			Melanoma sub-cohort	
	(*n* = 34)			(*n* = 85)	
Group	CTRL-NSCLC	NSCLC	MelVac-CTRL /MelVac	CTRL-Mel	PEM-Mel
Individuals	Controls without cancer (*n* = 10)^a^	NSCLC without MelCancerVac® therapy (*n* = 18)^b^	NSCLC with MelCancerVac^®^ therapy (*n* = 6)^c^	Healthy controls (*n* = 80)^a^	Melanoma patients with pembrolizumab therapy (*n* = 5)^a^
Age (mean ± SD)	65.3 ± 8.4	59.2 ± 7.9	55.7 ± 8.4	38.5 ± 10.7	67.6 ± 9.2
Gender (M/F/NA)	5/5/0	7/8/3	4/2/0	42/38/0	2/3/0
Samples	∑ samples = 130				

*NSCLC* – non-small cell lung cancer patients; *CTRL-NSCLC* – non-cancer controls for NSCLC group; *MelVac* – NSCLC patients who received MelCancerVac^®^ vaccine; *MelVac-CTRL* – paired samples of MelVac group taken before vaccination; *CTRL-Mel* – healthy controls for melanoma group; *PEM-Mel* – melanoma patients receiving pembrolizumab treatment; ^a^ – 1 sample per person available to researchers; ^b^ – 1 sample per person available to researchers, except for 3 patients (NSCLC1, NSCLC2, and NSCLC7) who had 2 samples available; ^c^ – 1 pre- and 1 post-vaccination sample of the patient available to researchers, except for one patient with 1 pre- and 3 post-vaccination samples. *F* – female, *M* – male*, n* – number of individuals; *NA* – not available.

The NSCLC patient cohort (*n* = 24) included longitudinal study of patients diagnosed with advanced NSCLC, who participated in the phase II clinical trial evaluating the effectiveness of MelCancerVac^®^ vaccine^38,39^ (Supplementary Table 2). The clinical trial, completed at the time of this study, was designed and carried out by Dandrit Biotech A/S and approved by European Medicines Agency (https://www.clinicaltrialsregister.eu/ctr-search/trial/2006-002202-54/DK). Out of the 24 study participants, 6 NSCLC patients donated blood samples before vaccination (group: *MelVac-CTRL*) and after receiving MelCancerVac® (group: *MelVac)*, while 18 NSCLC patients had not received any doses of the vaccine at the time of sample donation (group: *NSCLC*).

The melanoma group comprised of patients with unresectable and metastatic melanoma (*n* = 5, ICD-10: C43; group: *PEM-Mel*), who received KEYTRUDA^®^ (anti-PD-1 monoclonal antibody pembrolizumab, Schering-Plough Labo NV) immunotherapy as a part of standard-of-care. Serum samples of melanoma patients were collected 3 weeks after the first immunotherapy treatment, when patients came to receive the second dose (European Medicines Agency guidelines for KEYTRUDA therapy) and were provided by EGeen International (Mountain View CA, USA; ethical permit: 236/T-5).

Control groups included subjects with no history of cancer (*n* = 10, group: *CTRL-NSCLC*), with approvals for recruitment to the study from the Ethics Committee of the University Hospital of Liège (permit: 2018/77), and healthy blood donors (*n* = 80, ICD-10: Z52.0; group: *CTRL-Mel*) from the Blood Center of North Estonia Medical Center with the approval of the Ethics Review Committee on Human Research of the National Institute for Health Development, Estonia (permit: 1045).

### Mimotope variation analysis (MVA)

MVA, the next generation phage display method was used to determine individual immunoprofiles reflecting antibody repertoires for the study cohort^40,41^. Two µl of serum or plasma, previously precleared to plastic and E. coli/wt M13 phage particles, was incubated with 5 µl of phage library (~5 × 10^11^ phage particles, derivative of Ph.D.-12, NEB, UK) overnight at +4 °C. The human immunoglobulin G (IgG)-captured phages were pulled down by protein G-coated magnetic beads (NEB, S1506S). IgG-bound phage DNA was extracted and samples were barcoded and sequences amplified by PCR. Pooled samples were analyzed by Illumina sequencing (50 bp single end read, Brigham Young University DNA Sequencing Center, Utah, USA).

### MVA with DDM-1.7 cell line lysate competition

MelCancerVac® (DanDrit Biotech, Denmark/Enochian Biosciences, USA) is a therapeutic cellular vaccine based on autologous dendritic cells pulsed with the lysate of allogeneic melanoma cells (DDM-1.7) expressing several tumor antigens, including melanoma-associated antigens^42^. In MVA competition assay, freshly produced lysate from DDM-1.7 melanoma cells (Cellin Technologies, Estonia) was used to pre-block the study samples before MVA assay. Briefly, 30 µl of cell lysate (3 mg/ml) was incubated with 2 µl of serum or plasma before overnight incubation with the phage library and MVA was conducted as described.

### Data analysis and peptide antigen clustering

Data were processed with peptide data sets cleaned of sequencing errors and known artefacts, and counts normalized to 3 million reads^40,41^. Final dataset of 12-mer peptides consisted on average of 3.26 × 10^6^ peptide sequences (5.8 × 10^5^ unique) per sample, with a combined total of ~4.2 × 10^8^ peptide sequences. SPEXS2 exhaustive pattern search algorithm^40,41^ was used to group similar peptides and reveal enriched recognition patterns (epitopes) in the studied peptide sets (Supplementary Fig. 1a). Each sample was analyzed separately for identification of sample-specific epitopes that had ≥4 fixed amino acid positions. For data analysis of *MelVac* samples, the identification of epitopes was performed in a discriminative manner, where peptide sets from *MelVac-CTRL* and *MelVac* samples of the same patient were compared to each other. Epitopes that represented peptides that were at least 2-fold more enriched in the query sample (*MelVac*) as compared to paired sample peptide set (*MelVac-CTRL*) and with a hypergeometric *p*-value < 1 × 10^−8^ were selected for further analysis. For melanoma cohort (*n* = 5, *PEM-Mel*) the identification of epitopes was performed as non-discriminatory, where patient-specific epitopes were identified in comparison to a random-generated peptide set. Epitopes that represented peptides that were 10-fold more enriched in the query (*PEM-Mel*) than randomly generated reference peptide set and had a hypergeometric *p*-value < 1 × 10^−8^, were selected for further analysis. Altogether 54,055 core epitopes for melanoma and 18,021 epitopes for *MelVac* groups were selected, representing a dataset of melanoma-specific antibody immune response. In addition, pairwise comparison of *MelVac-CTRL* and *MelVac* sample datasets generated 17,690 pre-treatment-specific core epitopes.

### Sequence alignment

The set of melanoma-associated antigens used in sequence alignment were chosen from Weinert et al., 2009 data describing genes expressed in the DDM-1.7 melanoma cells^42^ (Supplementary Fig. 1b). Sequences of the epitopes of the antigens were downloaded from Immune Epitope Database (IEDB^43^, date accessed: 24.09.2020, www.iedb.org). Altogether, the IEDB database contained 2234 epitopes of 102 proteins expressed in the melanoma cell lysate DDM-1.7^42^. All antigen alignments were conducted using custom Excel VBA scripts.

For sequence similarity analysis, 2234 linear IEDB epitopes were exactly aligned with 54,055 melanoma and 18,021 vaccination-specific epitopes generated with SPEXS2. Thirty-five database entries (altogether 34 unique proteins) with sequence identity to at least 1 epitope from both melanoma and vaccination-specific epitope sets were recruited for further antigen-specific analysis. Primary protein sequences were downloaded from UniProtKB database^44^ using accession codes matching IEDB epitope entry names (date accessed: 09.10.2020, www.uniprot.org). These 35 protein sequences were aligned with 54,055 melanoma, 18,021 vaccination-specific, and 17,690 pre-vaccination-specific epitopes, with the criteria that every fixed amino acid from SPEXS2-determined epitopes was to match with the protein sequence. Out of these, altogether 8562 epitopes aligned to sequences of 35 melanoma-associated antigens.

### ELISA

Human cytomegalovirus (CMV) and Epstein-Barr virus (EBV) serostatuses were measured from blood samples with ISO-17025 accredited methods. In brief, serological analyses were performed with anti-CMV ELISA (IgG) method (EUROIMMUN EI 2570–9601G) and with anti-EBV-CA ELISA (IgG) method (EUROIMMUN EI 2791–9601G) according to the manufacturer’s specifications. Absorbance was measured at 450 nm with SpectraMax Paradigm (Molecular Devices). For CMV serology, 41 samples tested positive, 13 negative and 2 samples were borderline and therefore excluded from further correlation analyses. For EBV serology, all measured samples were conclusive: 35 tested positive, 3 samples were negative.

### Statistics and reproducibility

The study included 119 independent study subjects. Samples donated at different time points were considered as paired samples of the individual (*n* = 130). Technical replicates are defined as the same sample profiled in independent MVA experiments. No randomization or blinding to sample characteristics was conducted, samples were divided into groups based on clinically relevant diagnoses. Group-wise comparisons of median values were visualized using violin- or boxplots with individual data points, and statistical significance is shown where applicable. To evaluate the reproducibility of MVA data, the values of peptide abundance in two technical replicates were compared using Pearson’s correlation coefficient analysis (R package “ggpubr”) and the correlation value between replicates was established as *R* = 0.95 (*P* < 0.0001). Other samples were not measured repeatedly.

### Statistical analysis

Statistical analyses were conducted with R statistical programming language v.4.0.4 and RStudio environment v.1.4.1106^45,46^. Data were analyzed, graphs were produced and visualized using R packages “reshape2”, “tidyverse”, “precrec”, “ggpubr”, “ggsci”, “scales”, “patchwork”, “egg”, “ggalt” 2021 versions^45–58^.

Cosine similarity indices (CSIs) for sample comparisons based on top 2500 peptide abundance values and composition were calculated with the cosine function in R package “lsa”^59^.

Top 50 immunodominant characteristics were defined from group-specific epitopes generated in SPEXS2 analysis. For post- (*Vac*, *n* = 6) or pre- (*Pre*, *n* = 6) vaccination samples the abundance of group-specific epitopes (18,021 for *Vac* and 17,690 for *Pre*, respectively) were calculated as the number of IgG-bound peptides containing the epitope sequence in the sample. The 50 epitopes with the highest abundance values were selected for analysis. Z-scores for the comparison of antibody response to top 50 immunodominant characteristics were calculated individually for each patient. First, the mean of top epitope abundance values across both *Pre* and *Vac* samples was calculated, then the mean was subtracted from the value of each epitope (mean centered) and the result divided by the standard deviation (autoscaled). For graphical presentation the values are capped off at the 97.5th percentile value of each patient.

Boxplots were generated using the style of Tukey with R packages “ggpubr” or “ggplot2”^47,48^. In figures the upper, middle and lower boxplot lines represent the 75th, 50th, and 25th percentiles, while whiskers represent the largest or smallest value within 1.5 times interquartile range above the 75th percentile or below the 25th percentile, respectively. The *p*-values of two-sided Wilcoxon Rank Sum test were visualized with “ggpubr” or “ggplot2” packages^47,48^.

Wilcoxon Rank Sum test (with continuity correction, base R “stats” package^46^) was used to assess the group-differentiating features of 8562 unique epitopes aligning to melanoma-associated antigens, while custom Excel VBA script was used to determine the sensitivity and specificity while maximizing Youden’s index for each biomarker. MedCalc^®^ Statistical Software (v.19.7.2, www.medcalc.org; 2021) was used to conduct logistic regression and ROC analysis of 15 epitopes as a combinational test.

### Reporting summary

Further information on research design is available in the Nature Research Reporting Summary linked to this article.

## Results

### Highly individual patterns of top antibody response are elicited by immune therapy

**Fig. 1:**
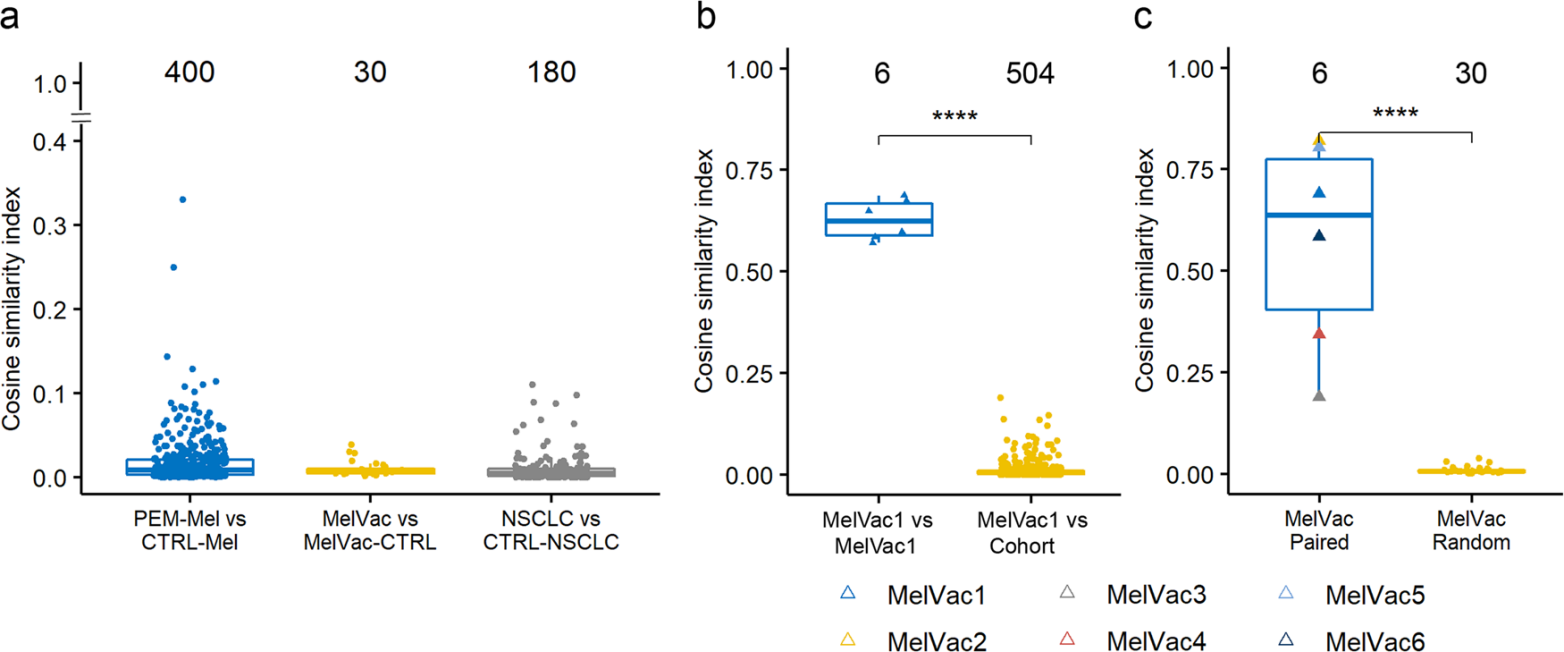
Top antibody response is individual-specific. **a**–**c** The top antibody response was analyzed using cosine similarity indices (CSI) by comparing the composition and abundance values of the 2500 most IgG-bound peptides in each sample to that of the rest of the cohort in pairs (Supplementary Data 1). CSI values (range from 0 to 1, *y-*axis) between samples belonging to the indicated groups (*x-*axis) are depicted. Numbers above boxplots indicate the number of comparison pairs shown as dots. Comparisons of samples to themselves (CSI = 1) are not depicted. Comparisons between different individuals are indicated with circles while, comparison of the samples of the same patient are indicated with triangles. **a** Pairwise comparison between study groups and their matched controls. *PEM-Mel* – melanoma patients receiving pembrolizumab treatment (n = 5); *CTRL-Mel* – healthy controls for melanoma group (*n* = 80); *MelVac* – NSCLC patients who received MelCancerVac® vaccine (*n* = 6); *MelVac-CTRL* – paired samples of MelVac group taken before vaccination (*n* = 6); *NSCLC* – non-small cell lung cancer patients (*n* = 18); *CTRL-NSCLC* – non-cancer controls for NSCLC group (*n* = 10). **b** Pairwise comparisons of the 4 longitudinal samples of one NSCLC patient, who received 35 doses of MelCancerVac® and remained with stable disease (Supplementary Table 2), to the 4 samples themselves (*MelVac1 vs MelVac1*) and to the rest of the study cohort (*n* = 126 samples, *MelVac1 vs Cohort*). **c** Pairwise comparisons of pre- and post-vaccination immunoprofiles of vaccinated NSCLC patients (*n* = 6). *MelVac Paired* – comparison of pre- and post-vaccination samples of the same patient; *MelVac Random* - comparison of the pre-vaccination sample of one patient to the post-vaccination samples of all 5 other patients. Two-sided Wilcoxon Rank Sum test, **** *p* < 0.0001, *p*-values not adjusted for multiple comparisons.

**Fig. 2:**
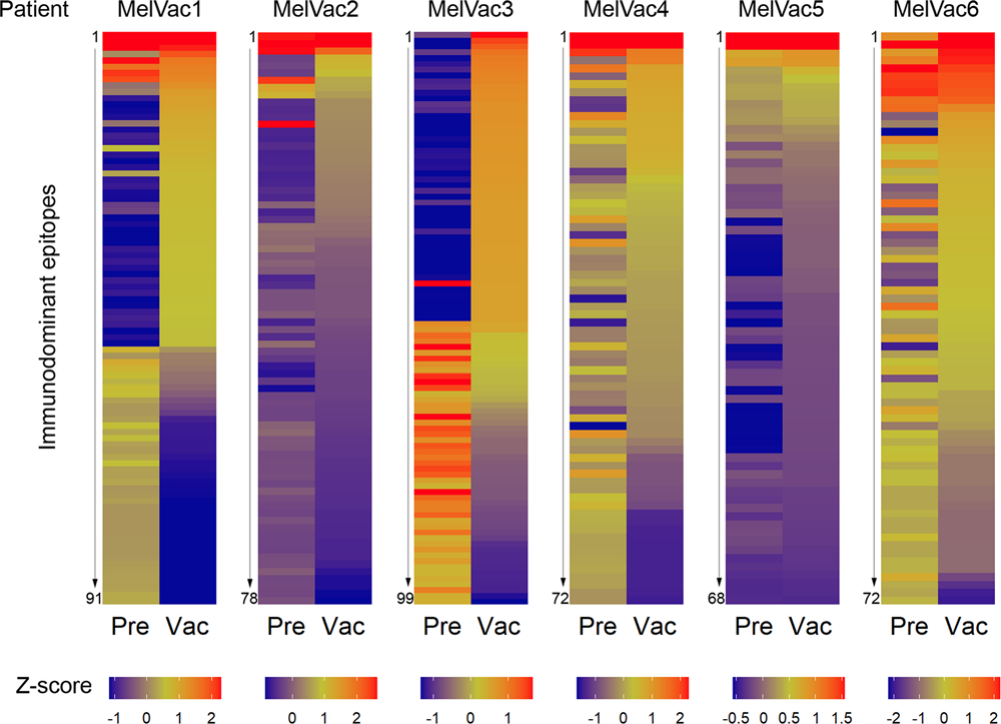
The heterogeneity of antibody response converges on immunodominant epitopes. Heatmaps depict differential antibody response to the 50 most immunodominant epitopes detected in pre- (*Pre*, *n* = 6) and post- (*Vac*, *n* = 6) vaccination samples of patients who received MelCancerVac® treatment (*MelVac1-MelVac6*). Rows depict immunodominant epitopes with numbers on the left of each panel referring to the specific epitope sequences provided in Supplementary Data 2. The number of epitopes differs for each patient as some epitopes were in the top 50 for both *Pre* and *Vac* samples, while some were detected in only one sample of the patient. Z-scores depict the abundance of IgG-binding peptides containing the immunodominant epitopes in each sample and are calculated separately for every patient by mean centering and autoscaling the abundance values across both *Pre* and *Vac* samples. Epitopes are ranked by highest-to-lowest abundance values as observed in the *Vac* sample. Z-score scale is cut-off at 97.5th percentile for better visualization of each panel.

To characterize immunotherapy-specific antibody repertoires and dissect the role of the immune system in biological therapies, our analysis included two different immunotherapy cohorts and controls comprising 119 individuals (Table 1 and Supplementary Table 1). First, sera samples of NSCLC patients from the phase II clinical trial receiving autologous DC vaccine MelCancerVac^®^ (Supplementary Table 2) and second, sera samples of melanoma patients receiving monoclonal anti-PD-1 antibody pembrolizumab as part of their standard care. To characterize the most prevalent antibody immune response in our study cohort, we used mimotope variation analysis (Supplementary Fig. 1)^40,41^. Briefly, individual blood samples were incubated with M13 phage-displayed 12-mer peptide library to capture individual-specific IgG antibody repertoires and high-throughput sequencing was used to uncover captured peptides. Cosine similarity index (CSI), a measure of similarity between the samples, was calculated to compare the top antibody response by analysis of seroresponse to 2500 peptides with the highest antibody reactivity in cohort samples (*n* = 130, Table 1). Analysis showed that different individuals with the same disease and immunotherapy background presented remarkable differences in the composition and magnitude of the dominant antibody response to different peptide antigens (CSI < 0.3, Supplementary Fig. 2a, Supplementary Data 1). The dominant antibody response to peptide antigens within groups of MelVac and MelVac-CTRL, PEM-Mel and CTRL-Mel was highly dissimilar (CSI < 0.4, Fig. 1a). However, longitudinal samples from the same individual showed similar IgG response to the top antigens (Supplementary Fig. 2b). For example, clear similarity in the top immunoprofile features of a patient denoted as MelVac1, who received 35 different vaccinations of MelCancerVac^®^ and showed stable disease over a 2-year period, was evident irrespective of the vaccination stage (CSI > 0.5, Fig. 1b). Overall, the antibody response profiles from the paired MelVac and MelVac-CTRL samples shared more similar features with each other than with unpaired NSCLC samples (Fig. 1c, Wilcoxon Rank Sum test, *p* < 0.0001, Supplementary Fig. 2b). Of note, the similarity to melanoma patients in the top 2500 peptide composition of NSCLC patients did not increase significantly upon vaccination with MelCancerVac^®^ (Wilcoxon Rank Sum test, *p* > 0.05, Supplementary Fig. 2c). Next, to delineate epitopes characteristic to MelVac group we used SPEXS2 exhaustive pattern search algorithm (Supplementary Fig. 1a). Comparison of seroresponse values to the top 50 most targeted epitopes in both pre- and post-vaccination samples of the same patient revealed common antigenic features present in both conditions (Fig. 2 and Supplementary Data 2). However, we also observed that seroresponse to the top 50 antigens changed upon MelCancerVac^®^ vaccination, although these changes were largely individual-specific (Fig. 2). Specifically, some patients maintained antibody reactivity to majority of epitopes upon vaccination (MelVac2, MelVac4–6), whereas the top response epitopes in others changed (MelVac1, MelVac3, Fig. 2). Overall, our data showed that even though intra-individual cancer immunoprofiles were more similar than inter-individual ones, the antibody immune response in NSCLC cohort was dynamic upon MelCancerVac® vaccination with changes both in the composition and abundance to immunodominant antigens.

### Immune reactivity targets epitopes of melanoma antigens

**Fig. 3:**
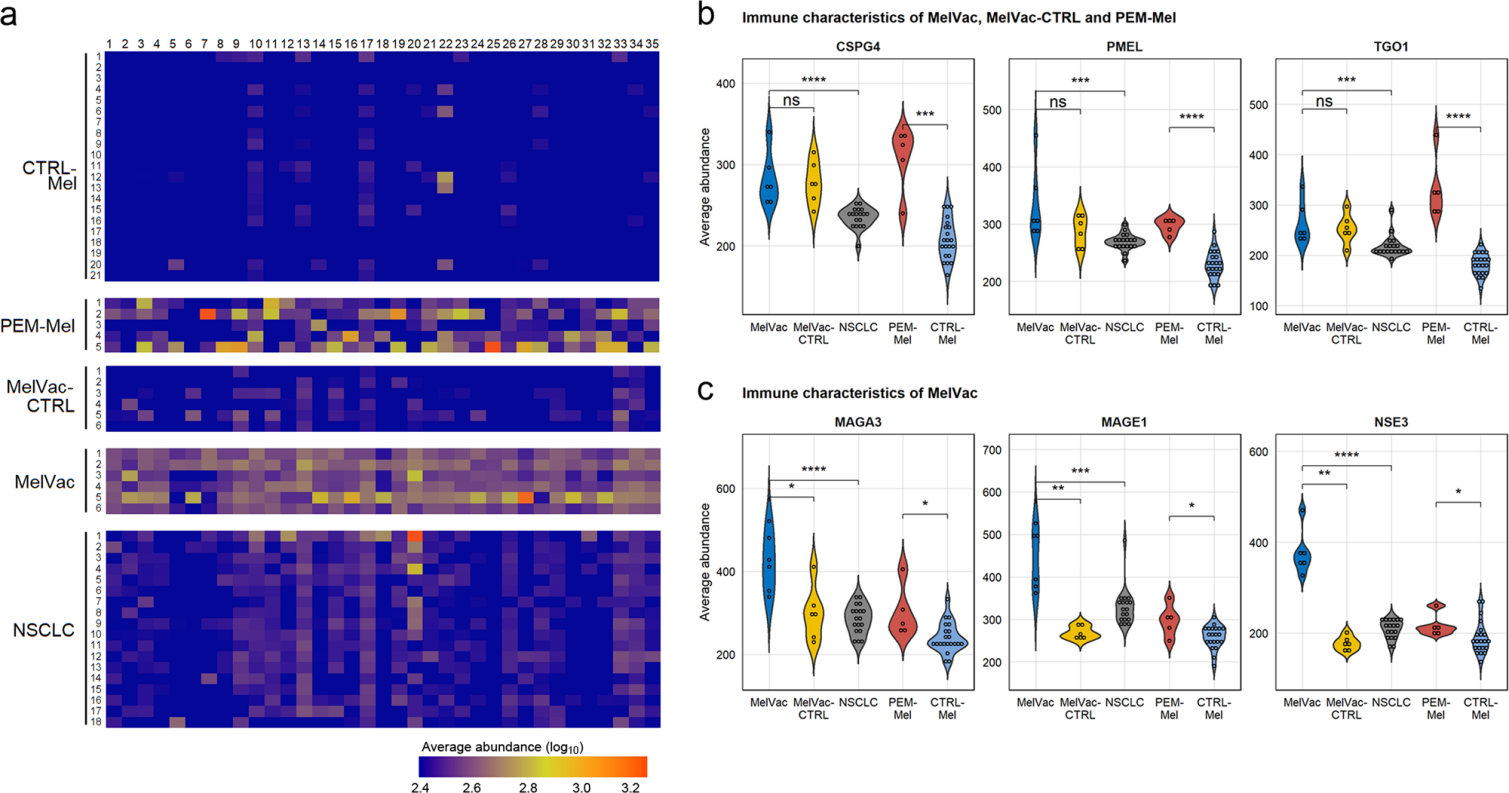
Individual-specific immunoprofiles of antibody response to melanoma-associated antigens. **a** Heatmap showing the antibody response to melanoma-antigens in patients with cancer and in the controls. Log_10_-transformation of the average abundance of the IgG-bound peptides containing epitopes with 100% identity to the indicated proteins are depicted. Value range ≤ 2.4 and ≥ 3.3 is provided for better visual representation. Group names of samples are depicted on the *y-*axis, individuals shown in numbers. *x-*axis indicates different melanoma-associated antigens (a total of 35 proteins) shown in Supplementary Table 3 and Supplementary Data 3. **b**, **c** Violin plots showing the average abundance of IgG-bound peptides containing epitopes with 100% identity to the specific proteins (shown above each graph, Supplementary Data 4) from panel **a** across study sub-cohorts. Two-sided Wilcoxon Rank Sum test, ns *p* > 0.5, *p < 0.05, **p < 0.01, *** *p* < 0.001, **** *p* < 0.0001, *p*-values not adjusted for multiple comparisons. *CTRL-Mel* – healthy controls for melanoma group (*n* = 21, all individuals older than 45 years); *PEM-Mel* – melanoma patients receiving pembrolizumab treatment (*n* = 5); *MelVac-CTRL* – paired samples of MelVac group taken before vaccination (*n* = 6); *MelVac* – NSCLC patients who received MelCancerVac^®^ vaccine (*n* = 6); *NSCLC* – non-small cell lung cancer patients (*n* = 18).

We hypothesized that dendritic cell vaccine therapy based on melanoma cell lysate could elicit melanoma-antigen-specific antibody response in NSCLC patients. In particular, considering that out of all protein antigens known to be expressed by the DDM-1.7 melanoma cells^42^, 102 proteins were reported to have epitopes showing serologically positive findings in Immune Epitope Database (IEDB). To characterize protein-specific immune responses in immunotherapy patients, we used SPEXS2 exhaustive pattern search algorithm to group individual peptides of MelVac, MelVac-CTRL, and PEM-Mel groups into representative epitopes and compared the delineated epitopes with known antigenic sequences. Altogether, antibody response to IEDB epitopes with sequence similarity to 35 proteins was detected in both MelVac and PEM-Mel groups (Supplementary Table 3). To characterize potential new antibody targets, we aligned the cancer-group-specific epitopes with the primary sequence of these 35 proteins. With an average of 340 epitope alignments per protein, 8562 unique epitopes from either MelVac, MelVac-CTRL or PEM-Mel group matched exactly with these antigens (Fig. 3a and Supplementary Data 3). Although, immunotherapy groups showed high reactivity to different sets of epitopes, the overall antibody response converged on the same antigenic proteins (Fig. 3a). Significant difference in antibody response to epitopes of known melanoma antigens CSPG4 (#4), PMEL (#6) and TGO1 (#31) was noticeable between PEM-Mel and MelVac and their respective control groups (Wilcoxon Rank Sum test, *p* < 0.001, Fig. 3b, Supplementary Data 4). Antibody response to a subset of melanoma-associated antigens (MAGA3 (#9), MAGE1 (#17), and NSE3/MAGEG1 (#25)) was pronounced in MelVac group and high in PEM-Mel group (Fig. 3c, Wilcoxon Rank Sum test, *p* < 0.05). Overall, we observed melanoma-associated antigen-specific IgG signatures elicited by MelCancerVac^®^ and anti-PD-1 immunotherapies.

### Fifteen group-discriminating epitopes converge on antigens associated with modulation of extracellular matrix and tumor cell survival pathways

To identify specific changes in immune response upon immunotherapy, we divided the study cohort into two subsets: controls (*n* = 90) and cancer samples with melanoma-associated attributes (*n* = 11, comprising of both MelVac and PEM-Mel groups). ROC analysis of 8562 antigen-associated epitopes resulted in 15 most group-discriminating antigenic determinants (markers M1 to M15) with sensitivity > 0.72 and specificity > 0.67 (Supplementary Fig. 3 and Supplementary Table 4). Notably, several of the resolved 15 biomarkers (M1-M15) mapped to the same antigens, but to different epitopes (M2 and M7 to MAGD2; M2 and M5 to MAGE1; M2 and M6 to PMEL; M3 and M11 to MORC4; M4 and M10 to MAGEMG50; M8 and M14 to CSPG4; M9 and M13 to CRGB1, Supplementary Table 5). Analysis of protein structure and biological relevance data from UniProtKB database indicated that majority of these 15 epitopes aligned to antigenic regions that were enriched in polar amino acids and located preferentially in regions with no known structural domains or in disordered segments. For example, M2 and M7 to MAGD2, M2, and M5 to MAGE1, M7 to MAGD1, M10 to MAGEMG50; M9 and M13 to CRGB1 and M12 to MAGE6. Some epitopes encompassed well-conformed domains like coiled-coil repeat of G3V599 for M1, leucine rich repeat of PRA22 for M3, cadherin-like CSPG repeats for M8 and M14. Biologically, these antigens are associated with extracellular-matrix formation (MAGEMG50) and modulation collagen protein turnover pathways (G3V599, CSPG4, and TGO1), with P53-associated apoptosis (MAGEMG50) and/or via ubiquitin ligase activity (MAGE1, MAGD1, MAGA3), but also with melanosome biogenesis (PMEL), ciliary signaling (ARMC) and lipoprotein signaling (G3V599). We also analyzed whether antibody response to common human herpesviruses, including cytomegalovirus (CMV) and Epstein-Barr virus (EBV) contributed to the treatment-elicited anti-cancer immunity given that EBV and CMV are the most prevalent infection types in tumors^60^ and can act as independent biomarkers for cancer immunotherapy^61^. For that, we analyzed EBV and CMV serology and showed that seropositivity to these common herpesviruses (Supplementary Fig. 4) was not correlated with treatment-elicited antibody response to the resolved top 15 melanoma-associated epitopes (Supplementary Data 5, 6). Based on both clinical serology and MVA data, we concluded that melanoma-associated antibody response linked to immunotherapy pointed onto apoptotic signaling and extracellular matrix-remodeling pathways conveyed by tumor-antigens but was not correlated with the common herpesviral antigens.

### MelCancerVac® boosts prior antibody response against a subset of melanoma-associated antigens

**Fig. 4:**
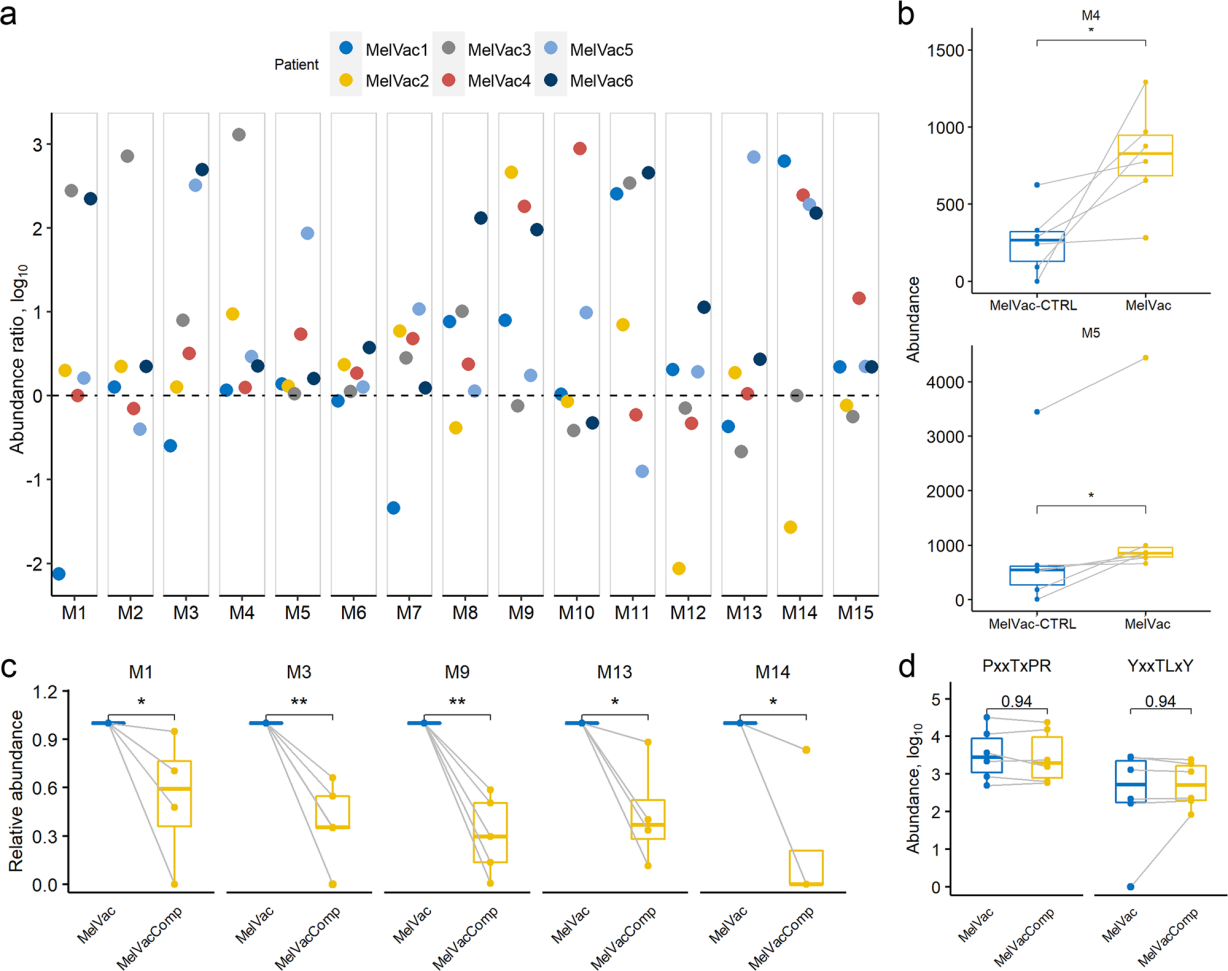
Antibody response to 15 melanoma-specific epitopes is pre-existing before MelCancerVac^®^ vaccination and boosted upon vaccine stimulation. **a** Comparison of antibody response to 15 epitopes in samples taken before (*n* = 6, *MelVac-CTRL*) and after vaccination (*n* = 6, *MelVac*) in six MelCancerVac® receiving patients (*MelVac1-MelVac6*). *x-*axis denotes 15 epitopes as biomarkers (*M1-M15*), *y-*axis (*Abundance ratio*) shows the ratio of abundance values of IgG-bound peptides between paired MelVac and MelVac-CTRL samples of the patient (MelVac_i_[*M*_abundance_ + 1]/MelVac-CTRL_i_[*M*_abundance_ + 1], *i* – number of patient, *M* – biomarker) in base 10 logarithmic scale. Dashed line indicates ratio value 0 (1 in linear scale), i.e., where antibody reactivity to peptides containing the specific epitopes remained unchanged in MelCancerVac® post-vaccination cohort. Values > 0 indicate rise in seroreactivity after vaccination while <0 indicates decrease. Source abundance values for each epitope are presented in Supplementary Data 5. **b** Vaccine-dependent antibody response enhancement to the resolved epitopes was common. Data are shown for epitopes M4 and M5 by comparing abundances of IgG-bound peptides from the vaccinated patients, before (*MelVac-CTRL*) and after vaccination (*MelVac*). *Abundance* – number of IgG-bound peptides containing the specified epitope sequence detected in the sample. Two-tailed paired Wilcoxon Rank Sum test, * *p* < 0.05, *p*-values not adjusted for multiple comparisons. **c** Box plots show the abundance of IgG-bound peptides containing the specified epitopes (M1, M3, M9, M13, and M14) upon MVA competition analysis. *MelVacComp* – data from competition with DDM-1.7 melanoma cell lysate is shown. *Relative abundance* – the abundance of IgG-bound peptides containing the specified epitopes normalized to values of the paired vaccination-specific sample (*MelVac*) for each patient. **d** Box plots show the abundance of IgG-bound peptides containing sequences of the viral capsid antigen p18 (EBV VCA p18 epitope (_161_ GGQPHDTAPRGARKK _175_) and the epitope of glycoprotein B (CMV gB; _70_ ETIYNTTLKY _80_)^40^ from MVA competition analysis. *MelVacComp* – data from competition with DDM-1.7 melanoma cell lysate is shown. *Abundance* – the abundance of IgG-bound peptides containing the specified epitopes in base 10 logarithmic scale.

Analysis of longitudinal samples revealed that antibody response to the majority of the 15 melanoma-differentiating epitopes was detectable in MelVac-CTRL samples with levels boosted by MelCancerVac^®^ administration in paired MelVac samples (Fig. 4a and Supplementary Fig. 5). Although the seroresponse changes to the majority of the 15 epitopes were patient-specific, reactivity to the epitope markers M4 (MAGEMG50) and M5 (MAGE1) was similarly boosted by vaccine in all patients (Wilcoxon Rank Sum test, *p* < 0.05, Fig. 4b). Furthermore, MVA with melanoma cell lysate competition confirmed that the high antibody reactivity observed in MelVac samples was specific to melanoma proteins as blocking with cell lysate interfered with IgG binding to most of the aforementioned epitopes, with significant effects for M1, M3, M9, M13, and M14 (Fig. 4c and Supplementary Fig. 6). The specificity of blocking with anti-melanoma-lysate was further confirmed by analyzing independent EBV and CMV-associated epitopes. Namely, the antibody response to epitopes of the viral capsid antigen p18 (EBV VCA p18; _161_ GGQ**P**HD**T**A**PR**GARKK _175_) and glycoprotein B (CMV gB; _70_ ETI**Y**NT**TL**K**Y**_80_)^40^ did not change upon competition (Fig. 4d). Therefore, we conclude that antibody response boosted upon MelCancerVac® vaccination is specific to epitopes related to melanoma-associated antigens and is frequently correlated with pre-existing immune response to these antigens.

### Three-epitope signature as a biomarker of immunotherapy-elicited melanoma-specific response

**Fig. 5:**
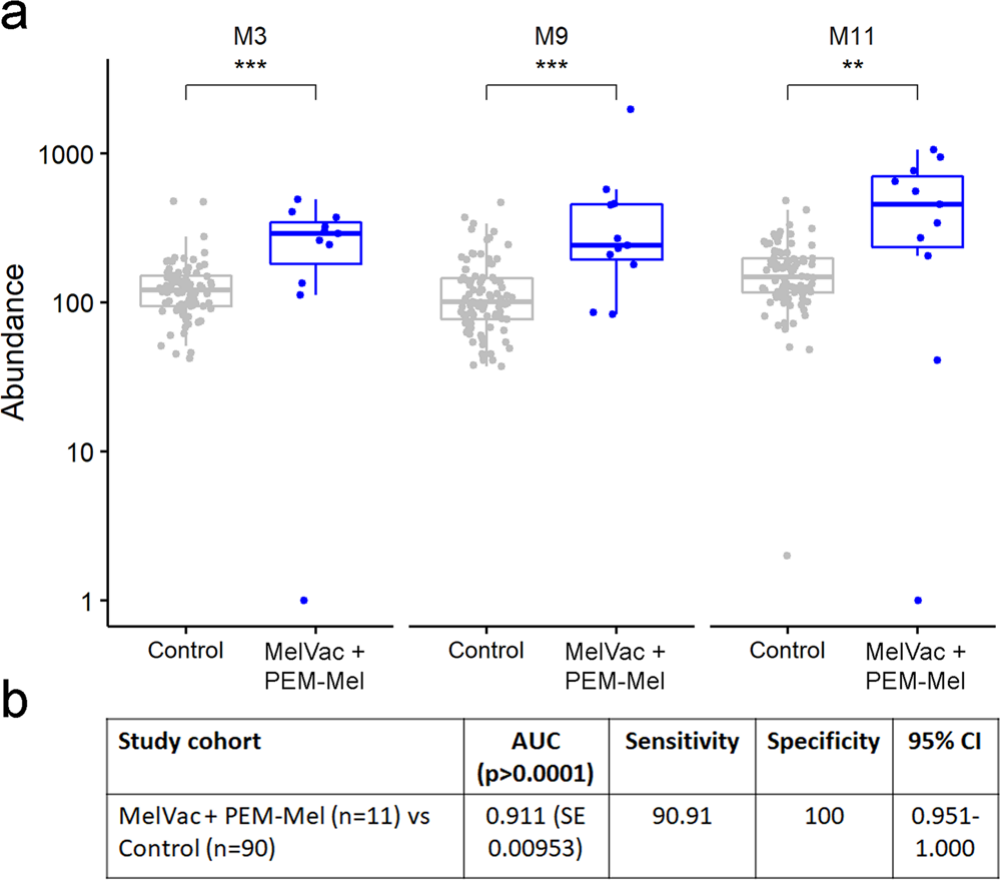
Antibody reactivity to epitopes of melanoma-antigens is associated with immunotherapy. **a** Box plots showing the IgG response to peptides containing the 3 epitopes that were most differentiating for melanoma-specific immunotherapy as deemed by logistic regression model analysis. *Abundance* – number of IgG-bound peptides containing the epitope biomarker sequence detected in a sample. *Control* – healthy controls for melanoma group (CTRL-Mel, *n* = 80) and non-cancer controls of NSCLC group (CTRL-NSCLC, *n* = 10); *MelVac* – NSCLC patients who received MelCancerVac^®^ vaccine (*n* = 6); *PEM-Mel* – melanoma patients receiving pembrolizumab treatment (*n* = 5). Two-sided Wilcoxon Rank Sum test **p < 0.01, *** *p* < 0.001, *p*-values not adjusted for multiple comparisons. **b** Logistic regression model of biomarkers M3, M9, and M11. AUC – area under curve; SE – standard error; CI – confidence interval.

As high melanoma-specific response appears to be a feature of tumor immunogenicity and its sensitivity to targeted treatments, we were interested to examine the prognostic utility of the resolved epitopes in therapy-elicited response. We used logistic regression and ROC analysis and found that 3 epitope biomarkers: M3 (ARMC9/PRA22/MORC4) (73% sensitivity, 97% specificity), M9 (CRBG1) (Sens 82% sensitivity, 81% specificity) and M11 (MORC4) (73% sensitivity, 90% specificity) in combination differentiated cancer patients based on melanoma-specific response elicited by treatments from controls with area under curve (AUC) of 0.991, ~91% sensitivity and 100% specificity (Fig. 5a, b and Supplementary Fig. 7). Here we show that antibody reactivity to a small subset of epitopes of known melanoma-associated antigenic determinants serves as a biomarker associated with melanoma-specific immunity elicited by cancer immunotherapies.

## Discussion

Here, we note unique and divergent changes in melanoma-antigen immune profiles in cancer patients receiving immunotherapy treatments that implicate distinct humoral immune functions connected to the therapy. The IgG response to specific epitopes of a subset of melanoma-antigens was associated with dendritic cell vaccine treatments in lung cancer. Patients receiving MelCancerVac^®^ showed prior response to some epitopes which was enhanced upon treatments, concluding these as potential biomarkers associated with anti-melanoma immunity already at the pre-treatment stage. The same epitopes were targeted by antibodies in melanoma patients receiving anti-PD-1 therapy. The resolved antigenic determinants were in proteins involved in the formation and modulation of extracellular matrix, and also tumor cell survival. Resolved antibody response targeting melanoma or melanoma-like features in cancer elicited by different types of immunotherapies sets the stage for future investigations of the epitopes and their clinical relevance as biomarkers for predicting therapy efficiency in larger studies.

Herein, we used MVA to discover epitope biomarkers associated with anti-cancer antibody response. Our data establish that individuals with cancer show highly heterogenous immune response to peptide antigens that is neither clinical group, cancer type nor immunotherapy specific. This finding was expected and could be related to the genetic variation and history of previously encountered pathogens^62^. However, we found clear similarities in antibody response to melanoma-associated proteins, including CTAs, a group of antigens now exceeding more than 200 proteins^63^ in patients upon different immunotherapy treatments. The resolved epitopes were mapped to extra- and intracellular proteins associated with the formation and modulation of extracellular matrix, but also with tumor cell survival, ciliary functions and lipoprotein signaling. Despite the fact that CTAs are mostly internal tumor antigens, the restricted expression of CTAs in tissues and their antigenicity has promoted utilizing them as targets for immunotherapies^64^. Interestingly, among other epitopes targeted by treatment-elicited antibody response in melanoma and NSCLC cases was of MAGE-A3 protein. MAGE-A3 has been detected in up to 76% of melanomas and in 30–50% of NSCLCs and is thus currently trialed as a target for immunotherapy^65,66^. Antibody response to epitopes coalescing on other MAGE group of proteins could be related to the poor prognostic features of metastases and melanoma progression^37,67^. On the other hand, given that the resolved group of antigens included widely expressed proteins (including MAGE family of antigens like MAGE-D), the humoral response towards these could mark excessive immune-attack and damage to self-tissues as concluded by others from studies of ICI-based therapies of melanoma and NSCLC^30,68,69^. This suggests that MVA-defined epitopes from intracellular antigens could be indicators of immunotherapy-associated tumor cell death. Our data on heterogenic immune response to melanoma-antigens are in good harmony with the findings on the heterogeneous expression of CTAs^70^. Therefore, characterization of the antibody response towards to canonical tumor antigens, including CTAs at pre- and post-treatment stage at epitope resolution could provide new strategies to detect and tackle cancer.

Most of the anti-melanoma-associated antigen immune response that we described for the NSCLC MelCancerVac^®^ cohort was also detected in patients with unresectable metastatic melanoma who received anti-PD-1 antibody (pembrolizumab) immunotherapy. We found that the resolved epitopes of MAGE-A antigens were similarly targeted by antibodies in both cases, suggesting the redundant cellular functions of the underlying antigens in lung cancer and melanoma. We expected to detect the anti-melanoma immunogenicity mainly in post-vaccination cases, but interestingly we determined antitumor immunity already at the pre-vaccine stage. Data from IFNγ analysis of MelCancerVac^®^ trial demonstrated T cell-specific response correlating with vaccine-specific immunity and sustained stable disease^38^. These findings on the ICI-associated restoration of T cell activity by MelCancerVac® are in good agreement with the observed anti-melanoma-specific humoral response patterns detected by our analysis. This once again highlights the importance to determine the elicited antibody response to specific tumor antigens as a measure of the anti-tumor activity associated with immune treatments for assessing the clinical utility of the treatment. However, to confirm the relationship between the melanoma-antigen associated epitopes and clinical efficacy, the number of patients needs to be substantially increased in future studies.

Autoantibodies have the potential to provide unique fingerprints that reflect the nature of the malignant process in the affected organ. Studies of B cell immunity on melanoma have demonstrated its important role in anti-tumor response, but also in irAEs associated with ICIs^71^. Relatedly, Toi et al. reported that although the presence of preexisting antibodies was associated with clinical benefits, it also led to the development of irAEs in NSCLC patients treated with anti-PD-1 monotherapy^69^. Therefore, the antibody epitopes we determined might be useful to assess immunopathological effects in order to minimize the probability of deleterious autoimmunity.

We validated the findings of epitopes mapped to melanoma-associated antigens from MVA competition analysis using melanoma cell lysate-specific antibody depletion approach from MelCancerVac^®^ samples. Interestingly, antibody response against three melanoma antigens observed in the pre-vaccination group, that were further confirmed to be therapy-connected by melanoma-lysate competition assay suggests that these could act as positive biomarkers of anti-tumor response to immune therapy. This is interesting in the light of the recent studies showing that preexisting antibody response in peripheral blood to tumor antigens, amongst these to MAGE1 carrying the resolved M5 epitope, is predictive of good clinical response to anti-PD-1 monotherapy of NSCLC^72–74^. Currently, high mutational burden in a tumor has been shown to predict sensitivity to anti-PD-1 therapies^75^. The expression of certain CTAs (for example MAGE-A) has been linked to poor disease prognosis due to reduced treatment response^76^ or is used to predict resistance to immunotherapy^77^. Our research on mapping the anticancer immunity to specific epitopes of tumor antigens prior to therapy could provide valuable prognostic knowledge for making the best therapeutic decisions, minimizing the probability of deleterious autoimmunity, and also for identification of novel targets for immunotherapy.

A big obstacle for effective cancer immunotherapy is the scarcity of immunotherapeutic biomarkers with specific clinical correlation to treatment efficacy and in relation to possible side effects. Here, we connected antibody profiles as defined by MVA to anti-tumor immunity elicited by immunotherapy at epitope precision. Our data provide support for the use of epitopes of tumor antigens as biomarkers for patient stratification and in immune monitoring. Future studies will elaborate on the connection between antibody profiles and ICI treatment outcomes.

There are several important caveats to this study. Although the MVA data covers a broad range of peptide antigens, extensive knowledge of the immune proteome of cancer is quite limited. Therefore, the mechanisms of how antibody response to the examined melanoma-specific antigens precisely impacts tumor immunogenicity, effective therapeutic targeting, or therapy efficacy remain to be solved. In addition, despite the use of multiple statistical methods and efforts to provide authentic targets to our findings, it is certainly possible for an epitope to play an important role in cancer and may mimic tumor-antigens but be derived from other antigens. For example, MAGE-A6 (amino acids 172–187) and *Mycoplasma penetrans* HF-2 permease (amino acids 216–229) share structural homology and elicit complex immunologic cross-reactivity^78^. We were thus careful with our conclusions: we can implicate an epitope by similarity to a self-protein but it is premature to exclude neoepitopes, cryptic epitopes, and metagenome-associated epitopes for which we do not have data. Finally, although this study harnessed the use of samples from a phase II clinical trial for the discovery of blood biomarkers, due to the limited number of samples further clinical studies are warranted.

## Supplementary information

Supplementary information

Description of Additional Supplementary Files

Supplementary Data 1

Supplementary Data 2

Supplementary Data 3

Supplementary Data 4

Supplementary Data 5

Supplementary Data 6

Reporting Summary

## Data Availability

Source data underlying the main figures are provided as Supplementary Data files 1–6. The whole sequencing datasets generated and/or analyzed in this study are not publicly available due to containing sensitive clinical information but are available from the corresponding author upon reasonable request via a material transfer agreement.

## Code availability

The R packages used to generate and analyze data presented in this study are described in the Methods section and available to all. The SPEXS2 algorithm for delineating epitopes from peptide sets can be found at https://github.com/egonelbre/spexs2. The custom Excel VBA used to find sequence similarities between generated epitopes and protein sequences are available from the corresponding author upon request.
