# Supplementary material for: Melanoma-specific antigen-associated antitumor antibody reactivity as an immune-related biomarker for targeted immunotherapies
Source: Commun Med (Lond). 2022 May 11;2:48. doi: 10.1038/s43856-022-00114-7 (PMC9095616; doi:10.1038/s43856-022-00114-7)
Supplement: Supplementary file 1 — Supplementary information [file 43856_2022_114_MOESM1_ESM.docx]

**Melanoma-specific antigen-associated antitumor antibody reactivity as an immune-related biomarker for targeted immunotherapies**

Annika Rähni^1,2^, Mariliis Jaago^1,2^, Helle Sadam^1,2^, Nadežda Pupina^1^, Arno Pihlak^1^, Jürgen Tuvikene^1,2,3^, Margus Annuk^4^, Andrus Mägi^5^, Tõnis Timmusk^1,2^, Amir M Ghaemmaghami^6^ and Kaia Palm^1,2*^

^1^Protobios Llc, Tallinn, Estonia;

^2^Department of Chemistry and Biotechnology, Tallinn University of Technology, Tallinn, Estonia;

^3^dxlabs Llc, Tallinn, Estonia;

^4^EGeen International Inc., Mountain View CA, USA;

^5^Tartu University Hospital, Tartu, Estonia;

^6^Immunology and Immuno-Bioengineering Group, School of Life Science, Faculty of Medicine and Health Sciences, University of Nottingham, Nottingham, United Kingdom

* Correspondence to Kaia Palm, kaia@protobios.com

**Supplementary Information**

**Supplementary** **Table 1. Overview of the samples used in this study from NSCLC patients participating in the MelCancerVac® vaccine phase II clinical trial and melanoma patients receiving anti-PD-1 monoclonal antibody (pembrolizumab) therapy.**

| Group | Patient sample | Immunotherapy | Number of samples | Time of sample collection | MVA competition  assay |
| --- | --- | --- | --- | --- | --- |
| MelVac-CTRL | MelVac-CTRL1 | - | 1 | Pre | - |
| MelVac | MelVac1 | MelCancerVac ® | 3 | Post: 6.5m (*), 14m, 24m | +, 6.5m sample |
| MelVac-CTRL | MelVac-CTRL2 | - | 1 | Pre | - |
| MelVac | MelVac2 | MelCancerVac ® | 1 | Post: 3w | + |
| MelVac-CTRL | MelVac-CTRL3 | - | 1 | Pre | - |
| MelVac | MelVac3 | MelCancerVac ® | 1 | Post: 2w | + |
| MelVac-CTRL | MelVac-CTRL4 | - | 1 | Pre | - |
| MelVac | MelVac4 | MelCancerVac ® | 1 | Post: 2w | + |
| MelVac-CTRL | MelVac-CTRL5 | - | 1 | Pre | - |
| MelVac | MelVac5 | MelCancerVac ® | 1 | Post: 3w | + |
| MelVac-CTRL | MelVac-CTRL6 | - | 1 | Pre | - |
| MelVac | MelVac6 | MelCancerVac ® | 1 | Post: 3w | + |
| NSCLC | NSCLC1 | - | 2 | Pre | - |
| NSCLC | NSCLC2 | - | 2 | Pre | - |
| NSCLC | NSCLC3 | - | 1 | Pre | - |
| NSCLC | NSCLC4 | - | 1 | Pre | - |
| NSCLC | NSCLC5 | - | 1 | Pre | - |
| NSCLC | NSCLC6 | - | 1 | Pre | - |
| NSCLC | NSCLC7 | - | 2 | Pre | - |
| NSCLC | NSCLC8 | - | 1 | Pre | - |
| NSCLC | NSCLC9 | - | 1 | Pre | - |
| NSCLC | NSCLC10 | - | 1 | Pre | - |
| NSCLC | NSCLC11 | - | 1 | Pre | - |
| NSCLC | NSCLC12 | - | 1 | Pre | - |
| NSCLC | NSCLC13 | - | 1 | Pre | - |
| NSCLC | NSCLC14 | - | 1 | Pre | - |
| NSCLC | NSCLC15 | - | 1 | Pre | - |
| NSCLC | NSCLC16 | - | 1 | Pre | - |
| NSCLC | NSCLC17 | - | 1 | Pre | - |
| NSCLC | NSCLC18 | - | 1 | Pre | - |
| PEM-Mel | PEM-Mel1 | Pembrolizumab | 1 | Post: 3w | - |
| PEM-Mel | PEM-Mel2 | Pembrolizumab | 1 | Post: 3w | - |
| PEM-Mel | PEM-Mel3 | Pembrolizumab | 1 | Post: 3w | - |
| PEM-Mel | PEM-Mel4 | Pembrolizumab | 1 | Post: 3w | - |
| PEM-Mel | PEM-Mel5 | Pembrolizumab | 1 | Post: 3w | - |

*NSCLC –* non-small cell lung cancer patients; *MelVac* – NSCLC patients who received MelCancerVac® vaccine; *MelVac-CTRL* – paired samples of *MelVac*; *PEM-Mel* – melanoma patients receiving pembrolizumab treatment; *Pre* – sample collected before immunotherapy; *Post* – sample collected after immunotherapy; *w* – weeks; *m* – months; * - vaccination-specific sample of MelVac1 represented in figures.

**Supplementary Table 2. Overview of the study subjects from the MelCancerVac® vaccine phase II clinical trial.**Detailed description of disease progression and tumor histology data in NSCLC patients who received MelCancerVac® vaccine as reported in Kvistborg et al. 2009 and Engell-Noerregaard et al. 2013^1,2^. Response to treatment was evaluated by CT scans after receiving 6^th^ and 10^th^ vaccine dose.

| Sample | Patients as referenced in MelCancerVac® clinical trial^1,2^ | Age | Gender | Vaccinations with MelCancerVac® | After 6th dose | After 10th dose | Histology |
| --- | --- | --- | --- | --- | --- | --- | --- |
| MelVac1/MelVac-CTRL1 | Patient no. 2 | 58 | M | 35 | SD | SD | SQ |
| MelVac2/MelVac-CTRL2 | Patient no. 4 | 57 | F | 6 | PD | - | AC |
| MelVac3/MelVac-CTRL3 | Patient no. 5 | 47 | M | 6 | PD | - | NOS |
| MelVac4/MelVac-CTRL4 | Patient no. 8 | 46 | M | 6 | PD | - | SQ |
| MelVac5/MelVac-CTRL5 | Patient no. 14 | 69 | M | 4 | PD | - | SQ |
| MelVac6/MelVac-CTRL6 | Patient no. 16 | 57 | F | 6 | PD | - | AC |
| NSCLC1 | Patient no. 1 | 66 | M | 26 | SD | SD | AC |
| NSCLC2 | Patient no. 6 | Excluded* |  |  |  |  |  |
| NSCLC3 | Patient no. 7 | 54 | F | 5 | PD | - | AC |
| NSCLC4 | Patient no. 10 | Excluded* |  |  |  |  |  |
| NSCLC5 | Patient no. 11 | 52 | F | 2 | - | - | SQ |
| NSCLC6 | Patient no. 12 | 68 | F | 16 | SD | PD | AC |
| NSCLC7 | Patient no. 13 | 58 | F | 10 | SD | PD | AC |
| NSCLC8 | Patient no. 17 | 50 | M | 5 | PD | - | AC |
| NSCLC9 | Patient no. 18 | 47 | M | 4 | PD | - | AC |
| NSCLC10 | Patient no. 19 | Excluded* |  |  |  |  |  |
| NSCLC11 | Patient no. 20 | 60 | M | 10 | SD | PD | AC |
| NSCLC12 | Patient no. 21 | 54 | F | 6 | PD | - | AC |
| NSCLC13 | Patient no. 22 | 62 | F | 6 | PD | - | NOS |
| NSCLC14 | Patient no. 23 | 67 | F | 6 | PD | - | AC |
| NSCLC15 | Patient no. 24 | 61 | M | 6 | PD | - | SQ |
| NSCLC16 | Patient no. 25 | 50 | M | 10 | SD | PD | NOS |
| NSCLC17 | Patient no. 27 | 65 | M | 6 | PD | - | NOS |
| NSCLC18 | Patient no. 28 | 74 | F | 5 | SD | - | AC |

*MelVac* – NSCLC patients who received MelCancerVac® vaccine; *MelVac-CTRL* – paired samples of MelVac group taken before vaccination; *NSCLC* – non-small cell lung cancer patients; *M* – male; *F* – female; *Excluded** – patients were excluded from clinical trial; *SD* – stable disease; *PD* – progressive disease; *SQ* – Squamous carcinoma; *AC* – Adenocarcinoma; *NOS* – unspecified non-small-cell lung cancer.


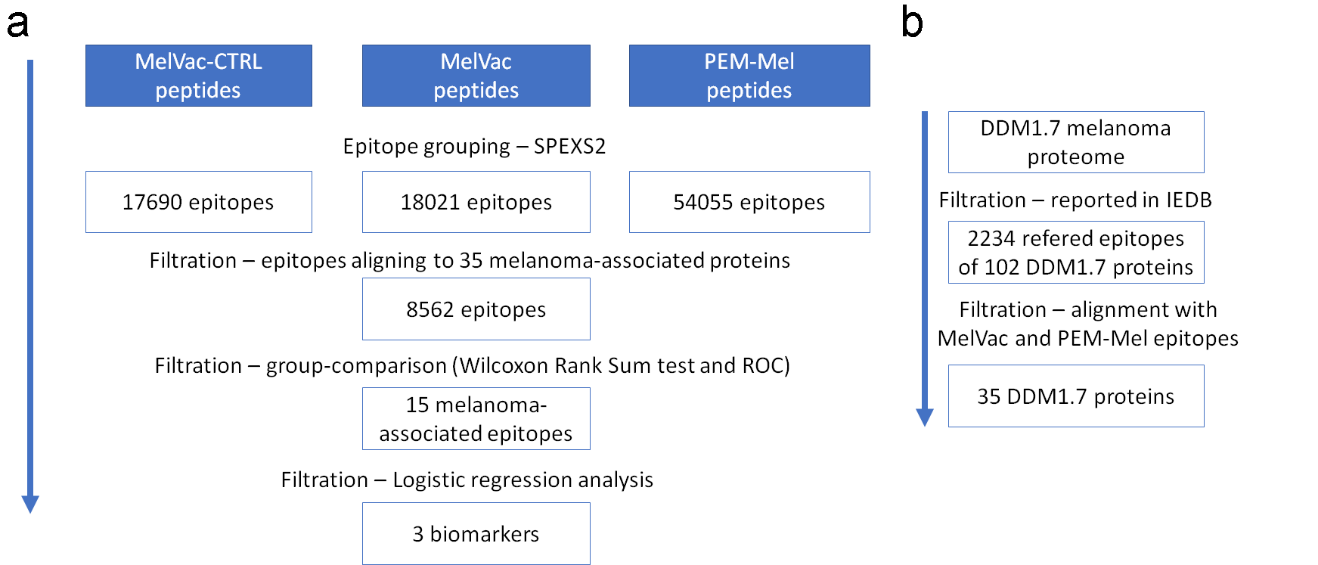


**Supplementary Figure 1. MVA data analysis workflow.**  MVA data analysis workflow scheme for delineating group-differentiating epitopes and melanoma-associated antigens (a) including the selection of melanoma-associated reference proteins and epitope alignments (b). *MelVac* – samples of NSCLC patients who received MelCancerVac®; *MelVac-CTRL* – paired samples of MelVac; *PEM-Mel* – samples of melanoma patients who received pembrolizumab treatments; *SPEXS2* – pattern search algorithm; *ROC* – receiver operating characteristic analysis; *DDM1.7* – melanoma cell line; *IEDB* – immune epitope database.


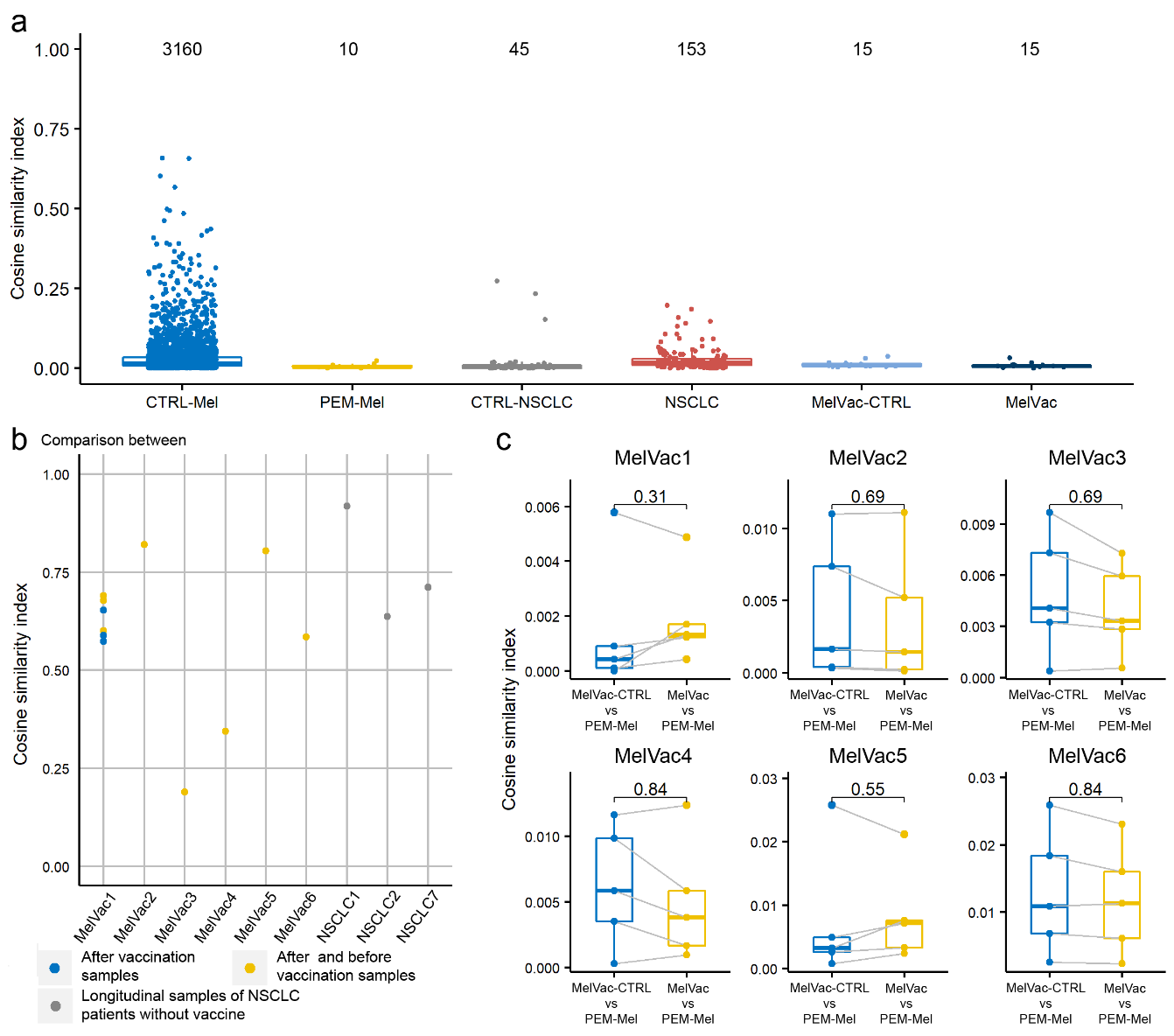


**Supplementary Figure 2. a-c| Comparison of antibody response to top 2500 peptide antigens in the study groups using CSI calculations, related to Figure 1. a|** The similarity (depicted as cosine similarity index values (CSI), *y-axis*) of antibody response to top 2500 peptide antigens between different individuals within the 6 study groups. *CTRL-Mel* – healthy controls for melanoma group (n=80); *PEM-Mel* – melanoma patients receiving pembrolizumab treatment (n=5); *CTRL-NSCLC* – non-cancer controls for NSCLC group (n=10); *NSCLC* – non-small cell lung cancer patients (n=18); *MelVac-CTRL* – paired samples of MelVac group taken before vaccination (n=6); *MelVac* – NSCLC patients who received MelCancerVac® vaccine (n=6). Numbers above boxplots indicate the number of pairwise comparisons shown as dots. **b|** The comparison of antibody response to top 2500 peptide antigens in longitudinal samples of NSCLC patients (*MelVac1-MelVac6*) taken before (*MelVac-CTRL*, n=6) or after (*MelVac*, n=6) MelCancerVac® vaccination or in longitudinal samples of 3 NSCLC patients (*NSCLC1-2, NSCLC7*) without immunotherapy. CSI values of pairwise comparisons of samples that were both taken after vaccination (*blue*) or before vaccination (*gray*), or of samples where one sample was taken before and other after vaccination (*yellow*). **c|** The comparison of antibody response to top 2500 peptide antigens between NSCLC patients who received MelCancerVac® vaccine and melanoma patients who received pembrolizumab immunotherapy. CSI values of pairwise comparisons of NSCLC samples from either before (blue, n=6) or after vaccinations (yellow, n=6) with melanoma samples from after pembrolizumab treatments (n=5). Two-tailed paired Wilcoxon Rank Sum test, p> 0.05, p-values not adjusted for multiple comparisons.

**Supplementary Table 3. Details of the 35 melanoma-associated antigens delineated in the current study.**

| Denotation in figures | Gene | Protein Name | Synonym | Protein Accession Code | Source | Number of IEDB epitopes in study |
| --- | --- | --- | --- | --- | --- | --- |
| 1 | ARMC9 | ARMC9 | KU-MEL-1 | Q7Z3E5 | sp | 36 |
| 2 | B2M | B2MG | B2MG | P61769 | sp | 463 |
| 3 | CRYBG1 | CRBG1 | CRBG1 | Q9Y4K1 | sp | 118 |
| 4 | CSPG4 | CSPG4 | CSPG4 | Q6UVK1 | sp | 92 |
| 5 | CTAG1A | CTG1B | CTA1/NY-ESO-1 | P78358 | sp | 32 |
| 6 | GAGE2D | GGE2D | GAGE2D/GAGE8 | Q9UEU5 | sp | 1 |
| 7 | PAGE2B | GGEE3 | PAGE2B | Q5JRK9 | sp | 3 |
| 8 | MAGEA1 | MAGA1 | MAGE1 | P43355 | sp | 82 |
| 9 | MAGEA3 | MAGA3 | MAGE3 | P43357 | sp | 56 |
| 10 | MAGEA10 | MAGAA | MAGE10 | P43363 | sp | 14 |
| 11 | MAGEA12 | MAGAC | MAGE12 | P43365 | sp | 15 |
| 12 | MAGEB2 | MAGB2 | MAGEB2 | O15479 | sp | 9 |
| 13 | MAGEC2 | MAGC2 | MAGEC2 | Q9UBF1 | sp | 18 |
| 14 | MAGED1 | MAGD1 | MAGED1 | Q9Y5V3 | sp | 64 |
| 15 | MAGED2 | MAGD2 | MAGED2 | Q9UNF1 | sp | 74 |
| 16 | MAGED4 | MAGD4 | MAGED4 | Q96JG8 | sp | 28 |
| 17 | MAGEE1 | MAGE1 | MAGEE1 | Q9HCI5 | sp | 15 |
| 18 | MAGEF1 | MAGF1 | MAGEF1 | Q9HAY2 | sp | 42 |
| 19 | MAGEH1 | MAGH1 | MAGEH1 | Q9H213 | sp | 18 |
| 20 | MLANA | MAR1 | MART1 | Q16655 | sp | 43 |
| 21 | MIA2 | MIA2 | MIA2 | Q96PC5 | sp | 71 |
| 22 | MORC2 | MORC2 | MORC2 | Q9Y6X9 | sp | 50 |
| 23 | MORC3 | MORC3 | MORC3 | Q14149 | sp | 45 |
| 24 | MORC4 | MORC4 | MORC4 | Q8TE76 | sp | 37 |
| 25 | NSMCE3 | NSE3 | MAGEG1 | Q96MG7 | sp | 28 |
| 26 | PMEL | PMEL | PMEL | P40967 | sp | 210 |
| 27 | PRAMEF22 | PRA22 | PRAMEF22 | A3QJZ6 | sp | 2 |
| 28 | PRAME | PRAME | PRAME | P78395 | sp | 50 |
| 29 | PXDN | PXDN | MAGEMG50 | Q92626 | sp | 28 |
| 30 | SS18 | SSXT | SSXT | Q15532 | sp | 43 |
| 31 | MIA3 | TGO1 | MIA3 | Q5JRA6 | sp | 162 |
| 32 | TPTE | TPTE | TPTE | P56180 | sp | 4 |
| 33 | MAGEA6 | E7ETG4 | MAGE6 | E7ETG4 | tr | 19 |
| 34 | SAGE1 | F5H2Z8 | SAGE1 | F5H2Z8 | tr | 2 |
| 35 | MIA2 | G3V599 | G3V599 | G3V599 | tr | 6 |

*sp* – UniProtKB/Swiss-Prot database*; tr* – UniProtKB/TrEMBL database; *IEDB* – immune epitope database


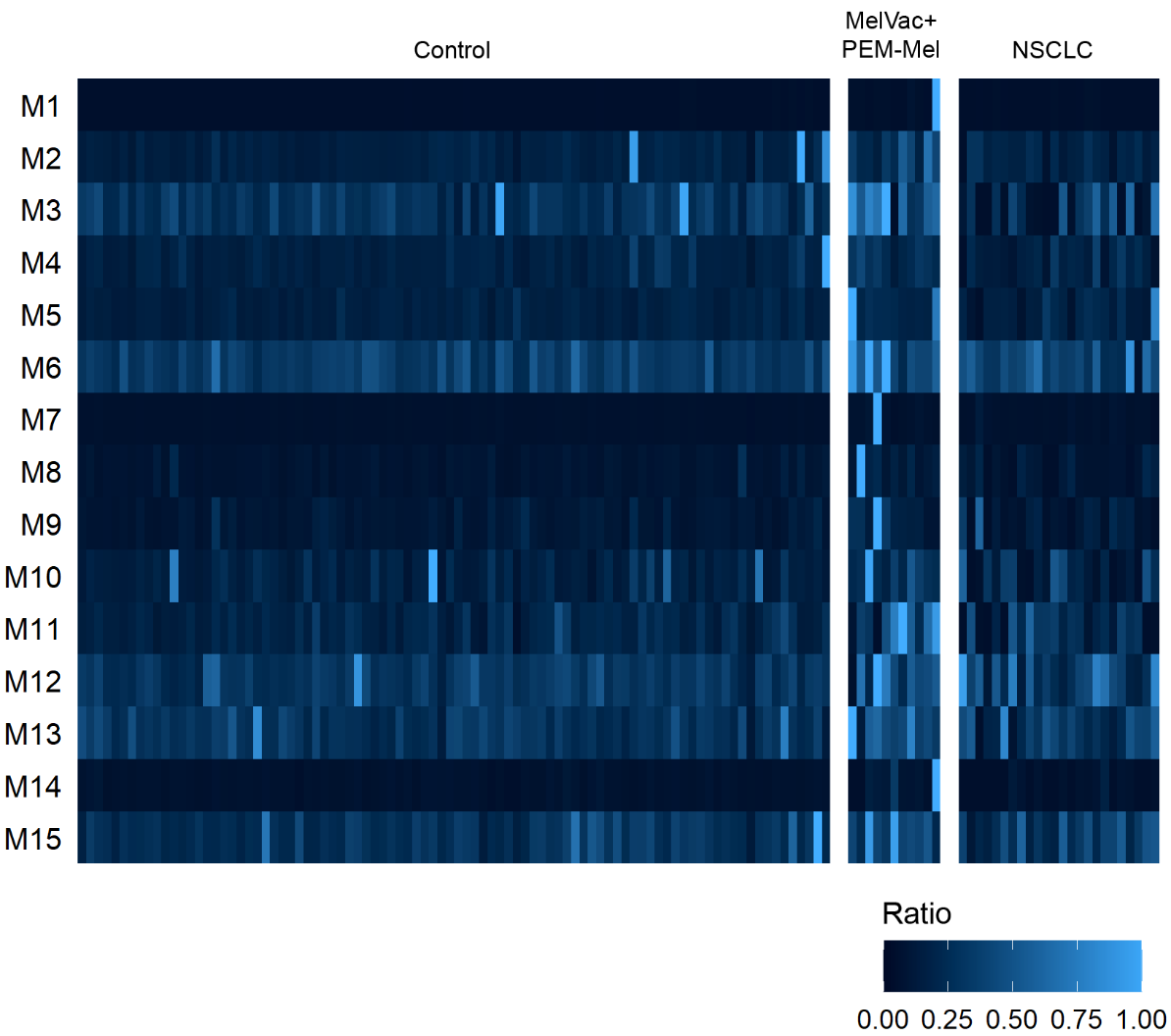


**Supplementary Figure 3. Antibody response to epitopes of melanoma-associated antigens discriminates between patients receiving immunotherapy treatments and non-cancer controls, related to Figure 4.** 15 epitopes (*M1-M15*) with discriminating IgG response between cancer patients and their matched controls (J statistic > 0.62, Sensitivity > 0.72, Specificity > 0.67). *Control* – cancer-free subjects (n=90, CTRL-Mel and CTRL-NSCLC groups); *MelVac* – NSCLC patients who had received MelCancerVac®(n=6); *PEM-Mel* – patients with melanoma who received pembrolizumab immunotherapy (n=5); *NSCLC* – non-small cell lung cancer patients not suited for immunotherapy (n=18) and MelVac-CTRL samples (n=6) (in total n=24); *Ratio* – abundance of IgG-bound peptides containing the specified epitope normalized to the highest value for the epitope across the study samples.

**Supplementary Table 4. ROC analysis of 15 melanoma-differentiating epitope biomarkers, related to Figure 4.** 15 highest scoring biomarkers (ranked by Youden’s J statistics) able to distinguish (J statistics > 0.6, Sensitivity >0.72, Specificity > 0.67) between PEM-Mel and MelVac cancer (n=11) and cancer-free (n=90). Sensitivity and specificity for each biomarker is calculated by maximizing Youden’s J statistic.

| Marker | Epitope | Threshold | True positives | False positives | Sensitivity | Specificity | J statistic |
| --- | --- | --- | --- | --- | --- | --- | --- |
| M1 | GxxxxxxPKxA | 110 | 8 | 5 | 0.73 | 0.94 | 0.67 |
| M2 | VPxxPxxS | 688 | 9 | 12 | 0.82 | 0.87 | 0.68 |
| M3 | VxxxxRDS | 216 | 8 | 3 | 0.73 | 0.97 | 0.69 |
| M4 | VPxxxPxxS | 618 | 9 | 9 | 0.82 | 0.9 | 0.72 |
| M5 | TxxxxPPT | 572 | 11 | 29 | 1 | 0.68 | 0.68 |
| M6 | TxxTPxxV | 474 | 10 | 20 | 0.91 | 0.78 | 0.69 |
| M7 | NAxxxxxTxP | 278 | 8 | 4 | 0.73 | 0.96 | 0.68 |
| M8 | VxxxxxQKP | 115 | 11 | 29 | 1 | 0.68 | 0.68 |
| M9 | KSPxxxxK | 166 | 9 | 17 | 0.82 | 0.81 | 0.63 |
| M10 | PxKxPxxG | 143 | 10 | 21 | 0.91 | 0.77 | 0.68 |
| M11 | MPxxxxRV | 257 | 8 | 9 | 0.73 | 0.9 | 0.63 |
| M12 | PPxxxxxxSS | 324 | 8 | 8 | 0.73 | 0.9 | 0.64 |
| M13 | SPxxxNxxxT | 382 | 9 | 17 | 0.82 | 0.81 | 0.63 |
| M14 | KxxxVxSF | 78 | 8 | 9 | 0.73 | 0.9 | 0.63 |
| M15 | QxxxxSRT | 454 | 8 | 9 | 0.73 | 0.9 | 0.63 |

*x* – any amino acid.

**Supplementary Table 5. Linking the Top15 epitope biomarkers with melanoma-associated antigens, related to Figure 3.**

| Marker | Epitope | Aligned protein ID | Gene | Synonym | Source | Alignment Start AA position | Target sequence |
| --- | --- | --- | --- | --- | --- | --- | --- |
| M1 | GxxxxxxPKxA | G3V599 | G3V599 | G3V599 | tr | 837 | GAYLDNPPKGA |
| M1 | GxxxxxxPKxA | Q96PC5 | MIA2 | MIA2 | sp | 910 | GAYLDNPPKGA |
| M2 | VPxxPxxS | Q9UNF1 | MAGD2 | MAGED2 | sp | 39 | VPETPKAS |
| M2 | VPxxPxxS | Q9HCI5 | MAGE1 | MAGEE1 | sp | 45 | VPQGPSDS |
| M2 | VPxxPxxS | F5H2Z8 | F5H2Z8 | SAGE1 | tr | 433 | VPNTPQIS |
| M2 | VPxxPxxS | P40967 | PMEL | PMEL | sp | 627 | VPQLPHSS |
| M3 | VxxxxRDS | Q7Z3E5 | ARMC9 | KU-MEL-1 | sp | 46 | VGGSFRDS |
| M3 | VxxxxRDS | A3QJZ6 | PRA22 | PRAMEF22 | sp | 359 | VDCGIRDS |
| M3 | VxxxxRDS | Q8TE76 | MORC4 | MORC4 | sp | 697 | VAKGVRDS |
| M4 | VPxxxPxxS | Q92626 | PXDN | MAGEMG50 | sp | 57 | VPAVAPQTS |
| M5 | TxxxxPPT | Q9HCI5 | MAGE1 | MAGEE1 | sp | 172 | TSTSVPPT |
| M6 | TxxTPxxV | P40967 | PMEL | PMEL | sp | 372 | TGMTPEKV |
| M6 | TxxTPxxV | P40967 | PMEL | PMEL | sp | 398 | TGMTPAEV |
| M7 | NAxxxxxTxP | Q9Y5V3 | MAGD1 | MAGED1 | sp | 99 | NAKDVPNTQP |
| M7 | NAxxxxxTxP | Q9UNF1 | MAGD2 | MAGED2 | sp | 110 | NADPQAVTMP |
| M8 | VxxxxxQKP | Q6UVK1 | CSPG4 | CSPG4 | sp | 564 | VILEHTQKP |
| M9 | KSPxxxxK | Q9Y4K1 | CRBG1 | CRBG1 | sp | 917 | KSPSHMEK |
| M10 | PxKxPxxG | Q92626 | PXDN | MAGEMG50 | sp | 1353 | PRKIPSVG |
| M11 | MPxxxxRV | Q8TE76 | MORC4 | MORC4 | sp | 592 | MPAPYRRV |
| M12 | PPxxxxxxSS | P43357 | MAGA3 | MAGE3 | sp | 61 | PPQSPQGASS |
| M13 | SPxxxNxxxT | Q9Y4K1 | CRBG1 | CRBG1 | sp | 652 | SPSSGNHLAT |
| M14 | KxxxVxSF | Q6UVK1 | CSPG4 | CSPG4 | sp | 953 | KTTMVTSF |
| M15 | QxxxxSRT | Q5JRA6 | TGO1 | MIA3 | sp | 1453 | QMMDVSRT |

*sp* – UniProtKB/Swiss-Prot database*; tr* – UniProtKB/TrEMBL database, *AA* – amino acid, *x* – any amino acid.


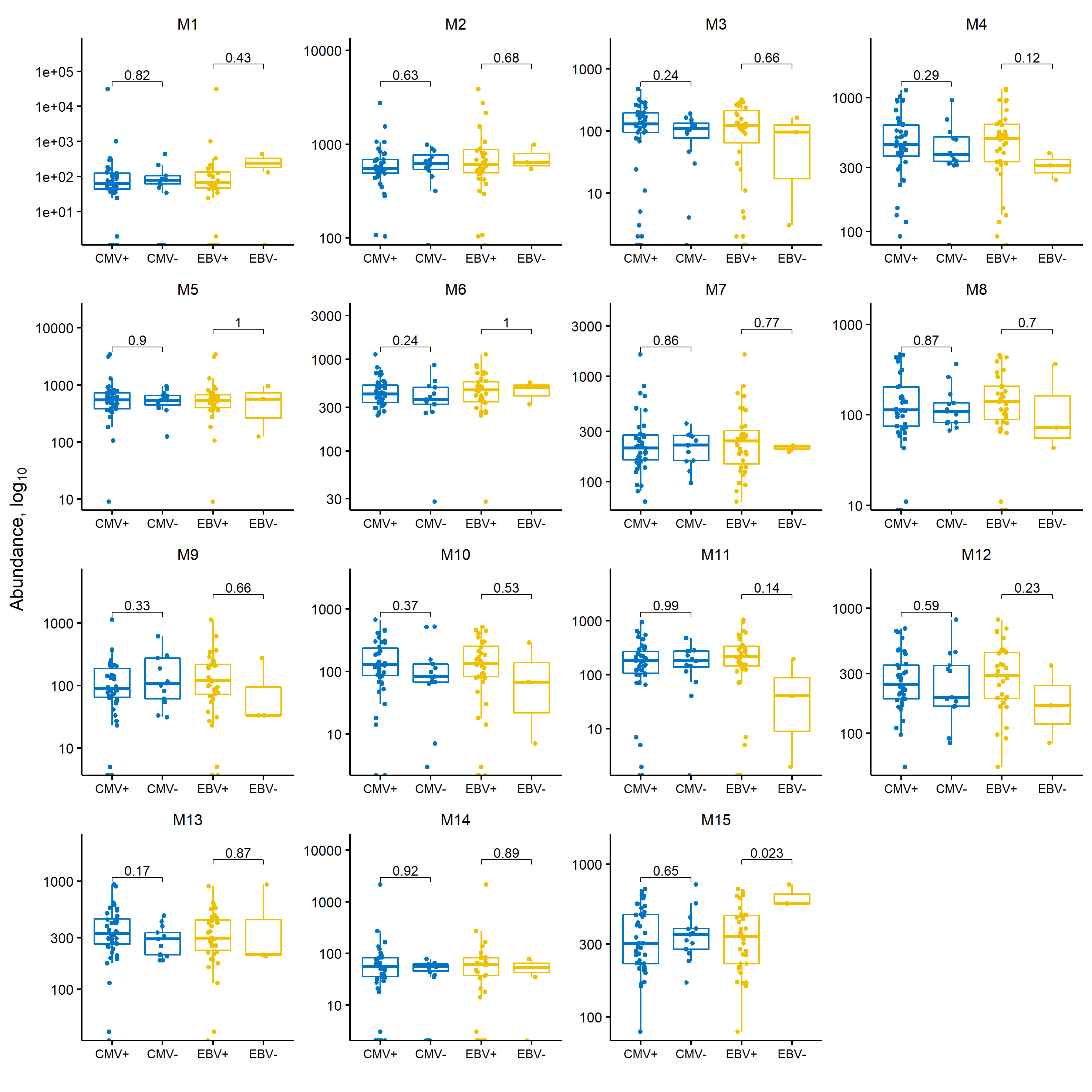


**Supplementary Figure 4. Seropositivity to CMV and EBV is not correlated with seroresponse to top 15 melanoma-associated epitope biomarkers**. Human cytomegalovirus (*CMV,* blue boxplots) and Epstein-Barr virus (*EBV*, yellow boxplots) seroreactivities were measured from blood samples with anti-CMV ELISA (IgG) (EUROIMMUN EI 2570-9601 G) and anti-EBV-CA ELISA (IgG) method (EUROIMMUN EI 2791-9601 G) according to the manufacturer’s specifications. *CMV+/EBV+* – samples positive for either anti-CMV (n=41) or anti-EBV-CA (n=35) IgG antibodies; *CMV-/EBV-* – samples negative for either anti-CMV (n=13) or anti-EBV-CA (n=3) IgG antibodies; *y-axis* – abundance of IgG-bound peptides containing specific marker sequences (*M1-M15*) in log_10_-transformation. Two-tailed Wilcoxon Rank Sum test, p-values not adjusted for multiple comparisons.


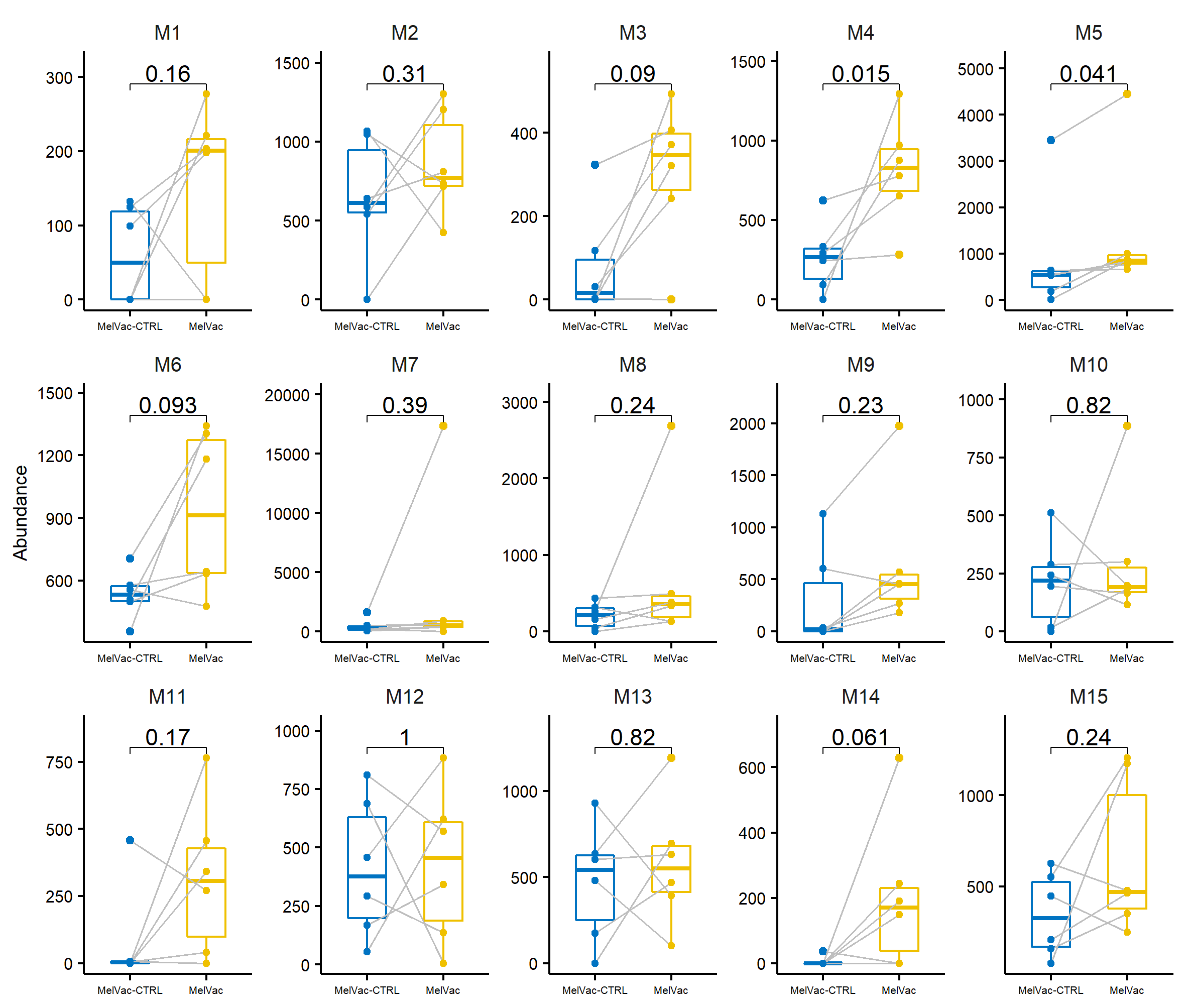


**Supplementary Figure 5. Antibody reactivity to peptides containing the top 15 epitopes in the vaccination cohort, related to Figure 5.** Group comparisons between vaccinated NSCLC cohort (n=6, *MelVac*) and their paired samples before vaccination (n=6, *MelVac-CTRL*). *Abundance* – number of IgG-bound peptides containing the specified epitope sequence in a sample. *Abundance* values of “0” i.e. “peptide not detected” are included in this pair-wise comparison. Two-tailed paired Wilcoxon Rank Sum test. p-values not adjusted for multiple comparisons.


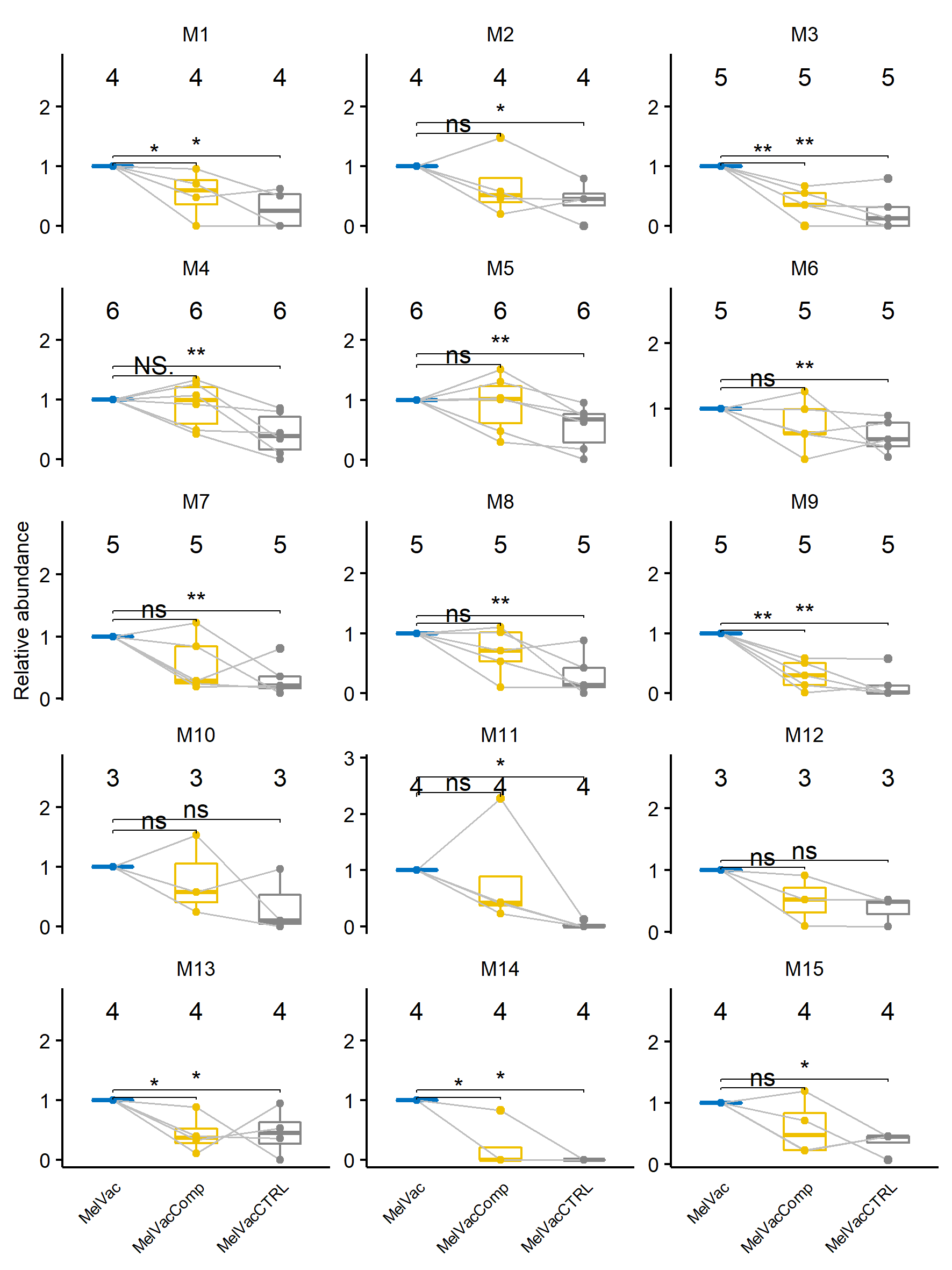


**Supplementary Figure 6. Anti-melanoma specific response to associated epitopes from MVA competition study, related to** **Figure 4.** *MelVac* samples were incubated with 30 µl of DDM-1.7 melanoma cell lysate before performing MVA analysis (*MelVacComp*) to block melanoma-specific IgG binding. Paired datapoints of the same patient are indicated with gray lines. Numbers on boxplots indicate the number of samples where marker abundance increased upon vaccination and that are represented on figure. *Relative abundance*, depicts the abundance of IgG-bound peptides containing the specified epitopes normalized to values in the paired vaccination-specific sample (*MelVac*) for each patient. *MelVac* – NSCLC patients who received MelCancerVac® vaccine; *MelVacComp* – MelVac sample with melanoma cell line lysate competition; *MelVacCTRL* – paired samples of MelVac group taken before vaccination. Two-tailed paired Wilcoxon Rank Sum test, ns p>0.5, *p<0.05, **p<0.01, p-values not adjusted for multiple comparisons.


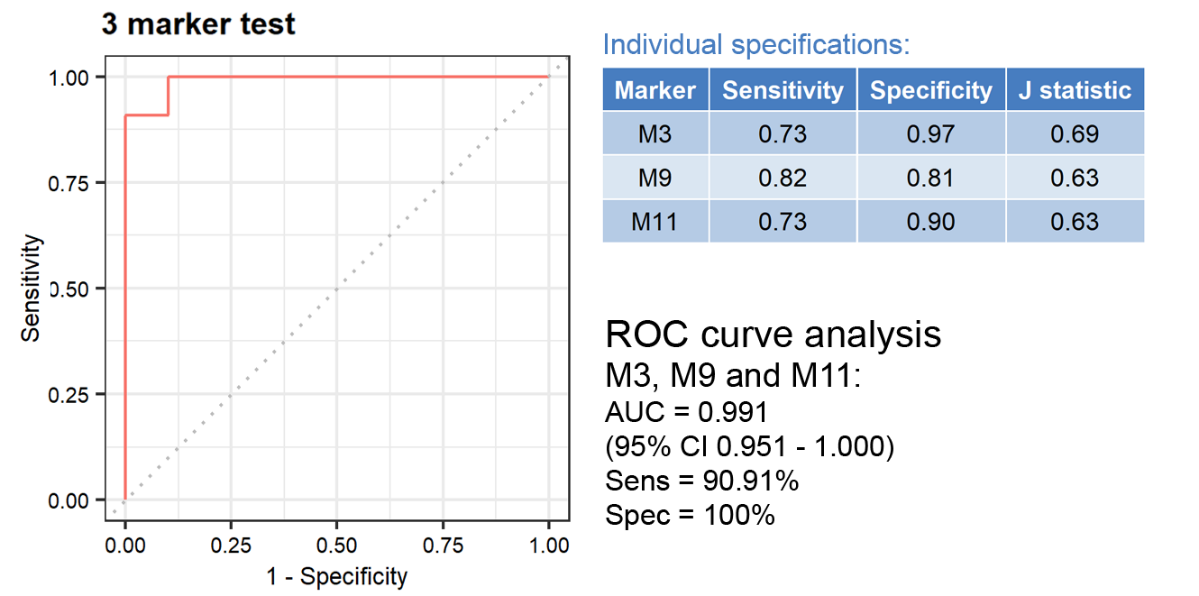


**Supplementary Figure 7. Logistic regression analysis of 15 biomarkers yielded in three epitope biomarkers (M3, M9 and M11) stratifying patients receiving immunotherapy (n=11) from controls (n=90), related to Figure 5.** Logistic regression analysis of 11 melanoma-associated samples (6 NSCLC patients who received MelCancerVac® and 5 melanoma patients who received pembrolizumab treatments) versus 90 non-cancer controls with following parameters: *method* – stepwise, *classification table cutoff value* = 0.5, *enter variable if P< 0.05*, *remove variable if P> 0.1*. Individual marker sensitivity, specificity and J statistic (*Individual specifications*) are from custom ROC analysis (**Supplementary Table 4**). ROC analysis of three biomarkers yielded AUC 0.991 (95% CI 0.951-1.000) with sensitivity 90.91% and specificity 100%. J statistic – Youden’s J score.; AUC – area under the curve, CI – confidence interval; Sens – sensitivity; Spec – specificity.

SUPPLEMENTARY REFERENCES

1. Engell-Noerregaard, L. *et al.* Clinical and Immunological Effects in Patients with Advanced Non-Small Cell Lung-Cancer after Vaccination with Dendritic Cells Exposed to an Allogeneic Tumor Cell Lysate. *World Journal of Vaccines* **3**, 68–76 (2013).

2. Kvistborg, P. *et al.* Comparison of monocyte-derived dendritic cells from colorectal cancer patients, non-small-cell-lung-cancer patients and healthy donors. *Vaccine* **28**, 542–547 (2009).
