# Supplementary material for: Melanoma-specific antigen-associated antitumor antibody reactivity as an immune-related biomarker for targeted immunotherapies
Source: Commun Med (Lond). 2022 May 11;2:48. doi: 10.1038/s43856-022-00114-7 (PMC9095616; doi:10.1038/s43856-022-00114-7)
Supplement: Supplementary file 2 — Description of Additional Supplementary Files [file 43856_2022_114_MOESM2_ESM.pdf]

Description of Additional Supplementary Files

File Name: Supplementary Data 1

Description: **Cosine similarity indices (CSI) for 130 samples, related to Figure 1.** CSI is calculated based on the count of 2500 most IgG binding peptide epitope abundance values in individual samples using R package "lsa". Groups: *PEM-Mel* – melanoma patients receiving pembrolizumab treatment (n=5); *CTRL-Mel* – healthy controls for melanoma group (n=80); *MelVac* – NSCLC patients who received MelCancerVac® vaccine (n=6); *MelVac-CTRL* – paired samples of MelVac group taken before vaccination (n=6); *NSCLC* – non-small cell lung cancer patients (n=18); *CTRL-NSCLC* – non-cancer controls for NSCLC group (n=10); *duplicate* – series samples of NSCLC or MelVac patients (n=5).

File Name: Supplementary Data 2

Description: **The highest seroresponse patterns at the epitope level for each MelCancerVac® receiving patient, related to Figure 2.** Seroresponse to 50 most abundant epitopes in pre- (*Pre*) and post-vaccination (*Vac*) samples for each NSCLC patient who received MelCancerVac® (*MelVac1-MelVac6*) are included along with the number of IgG-bound peptides for each epitope from mimotope variation analysis. The number of epitopes shown differs for each patient as some epitopes were detected among the top 50 of both, *Pre* and *Vac* samples. Epitopes are ranked by highest-to-lowest values as observed in the *Vac* sample. "x" in *Epitope* sequences denotes any amino acid.

File Name: Supplementary Data 3

Description: **Average abundance values of epitopes aligning to 35 melanoma-associated antigens, related to Figure 3a.** *CTRL-Mel* – healthy controls for melanoma group (n=21, all individuals older than 45 years); *PEM-Mel* – melanoma patients receiving pembrolizumab treatment (n=5); *MelVac-CTRL* – paired samples of MelVac group taken before vaccination (n=6); *MelVac* – NSCLC patients who received MelCancerVac® vaccine (n=6); *NSCLC* – non-small cell lung cancer patients (n=18).

File Name: Supplementary Data 4

Description: **Average abundance values of epitopes aligning to 35 melanoma-associated antigens, related to Figure 3b-c.** *CTRL-Mel* – healthy controls for melanoma group (n=21, all individuals older than 45 years); *PEM-Mel* – melanoma patients receiving pembrolizumab treatment (n=5); *MelVac-CTRL* – paired samples of MelVac group taken before vaccination

(n=6); *MelVac* – NSCLC patients who received MelCancerVac® vaccine (n=6); *NSCLC* – non-small cell lung cancer patients (n=18).

File Name: Supplementary Data 5

Description: **Abundance values of 15 melanoma-specific and 2 virus-related epitope containing peptides, related to Figures 4 and 5.** Groups: *PEM-Mel* – melanoma patients receiving pembrolizumab treatment (n=5); *CTRL-Mel* – healthy controls for melanoma group (n=80); *MelVac* – NSCLC patients who received MelCancerVac® vaccine (n=6); *MelVac-CTRL* – paired samples of MelVac group taken before vaccination (n=6); *NSCLC* – non-small cell lung cancer patients (n=18); *CTRL-NSCLC* – non-cancer controls for NSCLC group (n=10); *MelVacComp* – MelVac samples competed with DDM-1.7 melanoma cell lysate (n=6). *Marker* – biomarker M1 to M15 or virus-related epitopes (EBV or CMV). *Abundance* – number of IgG-bound peptides containing the specified epitope sequence detected in a sample.

File Name: Supplementary Data 6

Description: **ELISA measurements of CMV and EBV serology for cohort samples.** Human cytomegalovirus (CMV) and Epstein-Barr virus (EBV) serostatuses were measured from blood samples with anti-CMV ELISA (IgG) (EUROIMMUN EI 2570-9601 G) and anti-EBV-CA ELISA (IgG) method (EUROIMMUN EI 2791-9601 G) according to the manufacturer’s specifications. *NSCLC* – non-small cell lung cancer patients; *MelVac-CTRL* – samples of NSCLC patients taken before MelCancerVac® vaccination; *Control* – healthy controls for melanoma group; *PEM-Mel* – melanoma patients receiving pembrolizumab treatment; *POS* – sample positive for either anti-CMV or anti-EBV-CA IgG antibodies; *NEG* – sample negative for either anti-CMV or anti-EBV-CA IgG antibodies; *Borderline* – serology test inconclusive.
