# Supplementary material for: Melanoma-specific antigen-associated antitumor antibody reactivity as an immune-related biomarker for targeted immunotherapies
Source: Commun Med (Lond). 2022 May 11;2:48. doi: 10.1038/s43856-022-00114-7 (PMC9095616; doi:10.1038/s43856-022-00114-7)
Supplement: Supplementary file 9 — Reporting Summary [file 43856_2022_114_MOESM9_ESM.pdf]

nature research

Corresponding author(s): Kaia Palm, kaia@protobios.com

Last updated by author(s): Feb 28, 2022

Reporting Summary

Nature Research wishes to improve the reproducibility of the work that we publish. This form provides structure for consistency and transparency in reporting. For further information on Nature Research policies, see our Editorial Policies and the Editorial Policy Checklist.

Statistics

For all statistical analyses, confirm that the following items are present in the figure legend, table legend, main text, or Methods section.

n/a	Confirmed	
☐	☒	The exact sample size (n) for each experimental group/condition, given as a discrete number and unit of measurement
☐	☒	A statement on whether measurements were taken from distinct samples or whether the same sample was measured repeatedly
☐	☒	The statistical test(s) used AND whether they are one- or two-sided<br>*Only common tests should be described solely by name; describe more complex techniques in the Methods section.*
☐	☒	A description of all covariates tested
☐	☒	A description of any assumptions or corrections, such as tests of normality and adjustment for multiple comparisons
☐	☒	A full description of the statistical parameters including central tendency (e.g. means) or other basic estimates (e.g. regression coefficient) AND variation (e.g. standard deviation) or associated estimates of uncertainty (e.g. confidence intervals)
☒	☐	For null hypothesis testing, the test statistic (e.g. F, t, r) with confidence intervals, effect sizes, degrees of freedom and P value noted<br>*Give P values as exact values whenever suitable.*
☒	☐	For Bayesian analysis, information on the choice of priors and Markov chain Monte Carlo settings
☒	☐	For hierarchical and complex designs, identification of the appropriate level for tests and full reporting of outcomes
☐	☒	Estimates of effect sizes (e.g. Cohen's d, Pearson's r), indicating how they were calculated

Our web collection on statistics for biologists contains articles on many of the points above.

Software and code

Policy information about availability of computer code

Data collection	No software was used.
Data analysis	Data analysis was conducted with MedCalc® Statistical Software (v.19.7.2) and R Statistical programming language (v.4.0.4) in RStudio environment (v.1.4.1106).

For manuscripts utilizing custom algorithms or software that are central to the research but not yet described in published literature, software must be made available to editors and reviewers. We strongly encourage code deposition in a community repository (e.g. GitHub). See the Nature Research guidelines for submitting code & software for further information.

Data

Policy information about availability of data

All manuscripts must include a data availability statement. This statement should provide the following information, where applicable:

- Accession codes, unique identifiers, or web links for publicly available datasets
- A list of figures that have associated raw data
- A description of any restrictions on data availability

The data are not publicly available due to containing information that could compromise research participant privacy/consent. Any materials that can be shared will be released via a material transfer agreement.

Field-specific reporting

Please select the one below that is the best fit for your research. If you are not sure, read the appropriate sections before making your selection.

☒ Life sciences ☐ Behavioural & social sciences ☐ Ecological, evolutionary & environmental sciences

For a reference copy of the document with all sections, see nature.com/documents/nr-reporting-summary-flat.pdf

Life sciences study design

All studies must disclose on these points even when the disclosure is negative.

Sample size	Sample size was determined by the availability of blood samples from DanDrit Biotech phase II clinical trial and Egeen International Inc, Ca, USA.
Data exclusions	Based on previously conducted work, signals from peptides deemed as technical background and sequencing errors were excluded from MVA profiles before analysis.
Replication	Reproducibility of mimotope variation analysis was confirmed by establishing the correlation coefficient of two replicates as R=0.95. Other samples were not measured repeatedly.
Randomization	No randomization was conducted, samples were divided into groups based on clinically relevant diagnoses.
Blinding	Investigators were not blinded to study samples' origin or nature as data analysis was conducted specifically based on clinical grouping.

Reporting for specific materials, systems and methods

We require information from authors about some types of materials, experimental systems and methods used in many studies. Here, indicate whether each material, system or method listed is relevant to your study. If you are not sure if a list item applies to your research, read the appropriate section before selecting a response.

Materials & experimental systems

n/a	Involved in the study	
☒	☐	Antibodies
☐	☒	Eukaryotic cell lines
☒	☐	Palaeontology and archaeology
☒	☐	Animals and other organisms
☐	☒	Human research participants
☐	☒	Clinical data
☒	☐	Dual use research of concern

Methods

n/a	Involved in the study	
☒	☐	ChIP-seq
☒	☐	Flow cytometry
☒	☐	MRI-based neuroimaging

Eukaryotic cell lines

Policy information about cell lines

Cell line source(s)	The DDM-1.7 cell line was provided by DanDrit Biotech, Denmark (now Enochian Biosciences Inc., USA) and tested in Cellin Technologies LLC (Estonia).
Authentication	The expression of MAGE antigens A1, A2, A6, A10 and A12 with differentiating antigens of MART-1 and gp100 were used for authentication of DDM-1.7 cell line.
Mycoplasma contamination	Cell lines were not tested for mycoplasma contamination.
Commonly misidentified lines (See ICLAC register)	No misidentified lines were used in this study.

Human research participants

Policy information about studies involving human research participants

Population characteristics	The current study comprises blood samples from:<br>*5 patients diagnosed with unresectable and metastatic melanoma (ICD-10: C43), who received anti-PD-1 (pembrolizumab) immunotherapy<br>*24 non-small cell lung cancer patients who participated in a phase II clinical trial evaluating the effectiveness of MelCancerVac® vaccine

	*80 healthy blood donors (ICD-10: Z52.0) from North Estonia medical centre and 10 samples from donors with no medical history of cancer from CHU of Liège Centre.<br><br>The ages and gender proportions of groups are shown in Table 1 of the study.
Recruitment	Non-small cell lung cancer (NSCLC) patient samples were provided by DanDrit Biotech, Denmark, from the clinical trial approved by the Danish Medicines Agency and the local ethics committee (ClinicalTrials.gov with identifier NCT00442754, (see reference in Methods). Selected controls for NSCLC cohort are a part of PanBioRa consortium clinical study cohort and approved for use by the Ethics Committee of the University Hospital of Liège (permit: 2018/77). Melanoma patient samples were recruited with the approval of the Research Ethics Committee of the University of Tartu, Estonia (permit: 236/T-5) and provided by EGeen International Inc. CA, USA. The healthy control donor samples were were procured from Blood Center (North Estonia Medical Center) and approved by the Ethics Review Committee on Human Research of the National Institute for Health Development, Estonia.
Ethics oversight	See info in section "Recruitment" and in the Methods section of study.

Note that full information on the approval of the study protocol must also be provided in the manuscript.

Clinical data

Policy information about clinical studies

All manuscripts should comply with the ICMJE guidelines for publication of clinical research and a completed CONSORT checklist must be included with all submissions.

Clinical trial registration	This study was not a part of a clinical trial, but used samples from people previously enrolled in a phase II clinical trial (ClinicalTrials.gov with identifier NCT00442754).
Study protocol	No study protocol as this study was not a part of a clinical trial.
Data collection	Samples were collected in 3 different countries by 4 different institutions. The time of sample collection and data analysis is not relevant to study outcomes.
Outcomes	No outcomes as this study was not a part of a clinical trial.
